# The HIF-1/glial TIM-3 axis controls inflammation-associated brain damage under hypoxia

**DOI:** 10.1038/ncomms7340

**Published:** 2015-03-20

**Authors:** Han Seok Koh, Chi Young Chang, Sae-Bom Jeon, Hee Jung Yoon, Ye-Hyeon Ahn, Hyung-Seok Kim, In-Hoo Kim, Sung Ho Jeon, Randall S. Johnson, Eun Jung Park

**Affiliations:** 1 Cancer Immunology Branch, Department of System Cancer Science, National Cancer Center, 111 jungbalsan-ro, Kyunggi 410-769, Republic of Korea; 2 Department of Life Science, Hallym University, Chuncheon 200-702, Republic of Korea; 3 Department of System Cancer Science, Graduate School of Cancer Science and Policy, National Cancer Center, Goyang 410-769, Republic of Korea; 4 Department of Physiology, Development and Neuroscience, University of Cambridge, CB2 3EG Cambridge, UK

## Abstract

Inflammation is closely related to the extent of damage following cerebral ischaemia, and the targeting of this inflammation has emerged as a promising therapeutic strategy. Here, we present that hypoxia-induced glial T-cell immunoglobulin and mucin domain protein (TIM)-3 can function as a modulator that links inflammation and subsequent brain damage after ischaemia. We find that TIM-3 is highly expressed in hypoxic brain regions of a mouse cerebral hypoxia-ischaemia (H/I) model. TIM-3 is distinctively upregulated in activated microglia and astrocytes, brain resident immune cells, in a hypoxia-inducible factor (HIF)-1-dependent manner. Notably, blockade of TIM-3 markedly reduces infarct size, neuronal cell death, oedema formation and neutrophil infiltration in H/I mice. Hypoxia-triggered neutrophil migration and infarction are also decreased in HIF-1α-deficient mice. Moreover, functional neurological deficits after H/I are significantly improved in both anti-TIM-3-treated mice and myeloid-specific HIF-1α-deficient mice. Further understanding of these insights could serve as the basis for broadening the therapeutic scope against hypoxia-associated brain diseases.

## Abstract (web summary)

The targeting of inflammation has emerged as a promising therapeutic strategy for cerebral ischaemia. Here the authors show that TIM-3, a member of the T-cell immunoglobulin and mucin domain protein family, is upregulated in glial cells and plays a role in inflammation-associated brain injury under hypoxic conditions.

## Graphical abstract

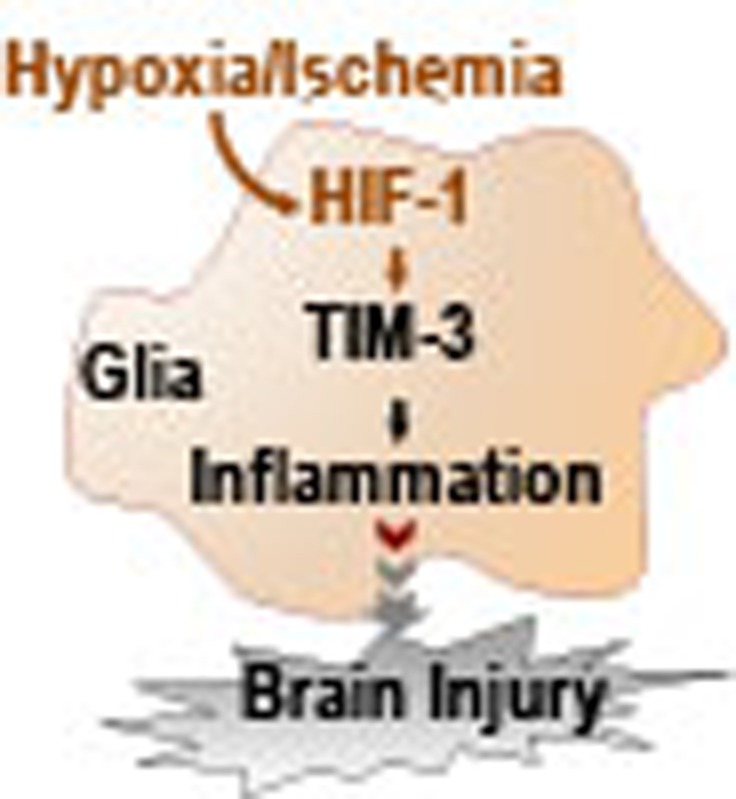

Cerebral ischaemia triggers a complex cascade of pathophysiological changes that ultimately lead to brain injury, particularly in the penumbral area surrounding the ischaemic core^1^,^2^. These alterations include the activation of resident cells, production of inflammatory mediators and infiltration of inflammatory cells. Clinical and experimental studies have shown that inflammatory interactions following brain ischaemia are closely related to the pathogenesis of brain injury, and strongly suggest that the inflammatory status might critically determine the outcome and prognosis of brain ischaemia^3^,^4^,^5^. In recent years, much attention has been focused on the therapeutic modulation of inflammatory status during cerebral ischaemia. However, the relevant information on the inflammatory events is very limited.

TIM-3, a member of the T-cell immunoglobulin and mucin domain protein family, was originally identified as a type 1 helper T cell (T_H_1)-specific surface molecule that negatively regulates T_H_1-dependent immune responses^6^. Subsequent studies have shown that TIM-3 is also expressed on multiple immune cell types, including T_H_17 cells, Tregs, NK cells, monocytes, dendritic cells, mast cells and microglia, where it potently regulates not only adaptive immunity but also innate immunity^7^,^8^,^9^,^10^. Recent studies have revealed that TIM-3 plays critical roles in regulating the activities of innate immune cells, functioning as either an activation marker or an activation limiter in a context-dependent manner^11^. TIM-3 has been closely associated with diverse immune-associated diseases, such as infection, autoimmune diseases and cancer, in both animal models and humans^6^,^12^,^13^,^14^. Interestingly, TIM-3 appears to have diverse functions under various pathological conditions, with its functional outcomes depending on the cell type and context^11^. For example, blocking of TIM-3 has been shown to improve the effector function of exhausted T cells in chronic viral infections and tumours^12^,^15^,^16^,^17^, whereas enhancement of TIM-3 signalling appears to ameliorate Th-1-mediated experimental autoimmune encephalomyelitis (EAE)^6^,^18^,^19^. Reduced levels of TIM-3 on CD4^+^CD25^−^ T cells reportedly contribute to impaired immunoregulation in autoimmune hepatitis^20^, whereas TIM-3 is overexpressed on CD4^+^ and CD8^+^ T cells in chronic hepatitis C infection^12^,^21^.

The physiological response to hypoxia is primarily mediated by hypoxia-inducible factor (HIF)-1, a heterodimeric transcription factor that consists of an oxygen-regulated α-subunit and a constitutive β-subunit^22^. The HIF-1 complex binds to the hypoxic-response elements (HREs) of many genes that have been linked with the adaptation to hypoxia^23^. Interestingly, HIF-1 can regulate cellular responses under not only hypoxic conditions but also inflammatory conditions, and plays an important role in the pathogenesis of several inflammation-associated diseases^24^,^25^,^26^,^27^. *In vivo* and *in vitro* experiments have demonstrated that HIF-1 is essential for myeloid cell-mediated inflammation such as myeloid cell motility^25^,^28^. In addition, HIF-1 activation has been implicated in pathogenic inflammatory responses after ischaemic lung and gut injuries^29^,^30^. Thus, HIF-1 is now considered to be a key regulator responsible for controlling inflammation-associated signalling events.

The central nervous system (CNS) has long been known to harbour immune-privileged regions, but recent work has shown that it is also equipped with an elaborate sentinel system that can rapidly trigger innate and subsequent adaptive immune responses^31^. Glial cells, which act as major immune cells in the immune responses of the CNS, recognize subtle changes in the brain and quickly respond to pathophysiological stimuli^32^,^33^. In this paper, we suggest that expression of TIM-3 on microglia and astrocytes is upregulated under hypoxia, and that this enhancement influences the infiltration of neutrophils into the hypoxic penumbra. Such infiltration has been identified as a main cause of ischaemic brain damage^5^,^34^. In addition, we show that HIF-1 controls the oxygen-dependent expression of TIM-3 in glial cells, and that not only TIM-3 blockade but also HIF-1 deficiency significantly improves functional neurological outcomes in mice. Collectively, our results suggest that hypoxia-induced glial TIM-3 may be an important molecular player in inflammation-associated brain injury under hypoxic conditions. These insights into the link between inflammation and ischaemic brain injury improve our understanding of the functions of glial TIM-3 and HIF-1, and may contribute to the development of new therapeutic strategies for cerebral ischaemia.

## Results

### TIM-3 expression is distinctly elevated in hypoxic penumbra

**Figure 1:**
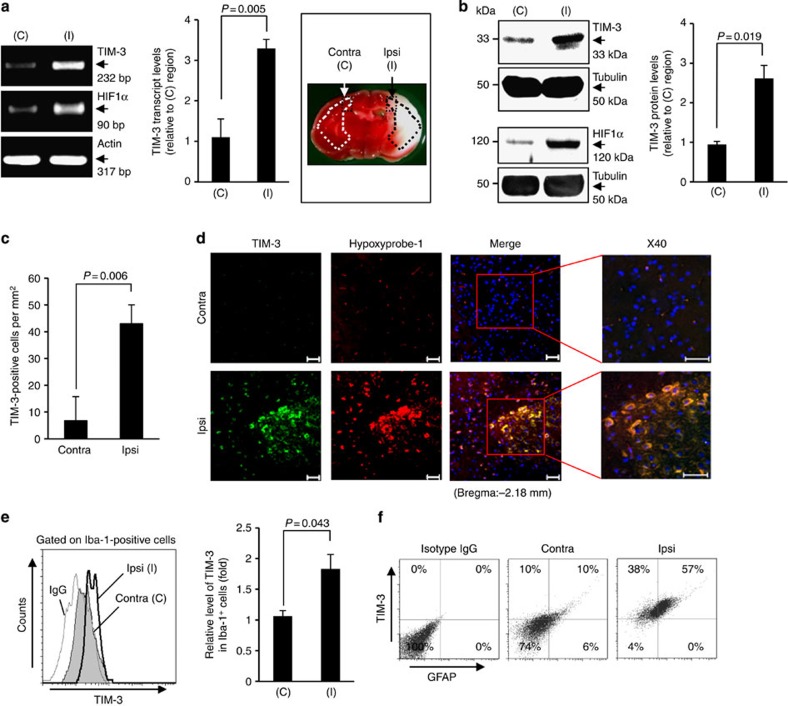
TIM-3 is highly expressed in hypoxic brain regions of a H/I mouse model. (**a**) TIM-3 transcript levels were examined in brain tissues from the contralateral cortex (C, boxed region) and ischaemic ipsilateral cortex (I, boxed region) of mouse model 24 h after H/I. The RT–PCR products were quantified with Image J and normalized with respect to the expression of actin. The HIF-1α transcript level represents a positive control for hypoxia. The right panel shows representative TTC staining of three brain sections from the H/I mice. (**b**) Representative western blot analyses of the TIM-3 and HIF-1α proteins (*n*=3). Relative levels of TIM-3 are shown as the mean±s.d. from three independent experiments. (**c**) Contralateral and ipsilateral cortical regions of coronal sections from the H/I mice were subjected to immunohistochemistry using an anti-TIM-3 antibody, and the number of TIM-3-expressing cells per mm^2^ was counted. (**d**) Immunohistochemistry was performed on brain sections from the H/I mice using anti-TIM-3 and hypoxyprobe-1 (red, to detect hypoxic regions). Scale bars, 50 μm ( × 20); 50 μm ( × 40). (**e**,**f**) Brain cells were isolated from the ipsilateral and contralateral hemispheres of three mice per group, processed for simultaneous detection of TIM-3 plus Iba-1 (**e**) or GFAP (**f**), and analysed by FACS. The results are presented as relative TIM-3 levels in the indicated gated populations, as determined from three independent experiments.

To examine the molecular mechanisms underlying the interdependent association between ischaemic brain damage and inflammation, we explored candidate molecular players that could exert key roles in the pathophysiological inflammatory events that follow cerebral hypoxia-ischaemia (H/I). For this, we utilized an experimental mouse model of transient unilateral cerebral ischaemia by unilateral ligation of the right carotid artery followed by exposure to systemic hypoxia^35^. Twenty-four hours after H/I, we obtained tissues from both contralateral and penumbral cortex regions (Supplementary Fig. 1), and then examined the expression levels of various inflammation-associated molecules at the RNA and protein levels. Interestingly, we found that transcript levels of T-cell immunoglobulin and mucin domain-3 (TIM-3) were notably higher in ipsilateral penumbra compared with contralateral regions. TIM-3 protein was also elevated in the ipsilateral penumbra, where the transcript and protein levels of HIF-1, a positive control that is reportedly abundant under hypoxia^36^,^37^, were increased compared with contralateral regions (Fig. 1a,b).

To confirm these results, we performed immunohistochemistry on coronal sections of H/I mice using an antibody against TIM-3 (refs 38, 39). Consistent with the above results, TIM-3-positive cells were markedly elevated in ipsilateral penumbra (Fig. 1c). Using the hypoxia marker pimonidazole (hypoxyprobe-1), we further demonstrated that TIM-3 was highly expressed in the hydroxyprobe-1-stained hypoxic penumbra of H/I mice (Fig. 1d). Taken together, these findings suggest that TIM-3 expression is upregulated in hypoxic penumbra, and imply that TIM-3 may play a role in the pathophysiological events following cerebral ischaemia.

### Glial TIM-3 is upregulated under hypoxia

We next examined which cells show TIM-3 upregulation after H/I. Western blot analyses revealed that protein expression levels of ionized calcium-binding adaptor molecule-1 (Iba-1, an activated microglial marker) and glial fibrillary acidic protein (GFAP, an activated astrocyte marker) were higher in the ipsilateral cortex than in the contralateral cortex of H/I mice at 24 h post-H/I, whereas the expression levels of neuronal cell markers, such as neuronal nuclei (NeuN), microtubule-associated protein 2 and glutamate decarboxylase, were reduced in penumbral cortex tissues (Supplementary Fig. 2a). We thus examined the expression levels of TIM-3 in microglia and astrocytes 24 h after H/I. Immunohistochemistry showed that a large portion of TIM-3-expressing cells in the ipsilateral cortex of the H/I mice were Iba-1 positive (Supplementary Fig. 2b). Strong TIM-3 expression was also observed in GFAP-immunoreactive astrocytes in the ipsilateral cortex.

Fluorescence-activated cell sorting (FACS) analyses of the brain cells isolated from the H/I mice further showed that hypoxia-ischaemia results in activation of microglia and astrocytes, which exhibit elevated TIM-3 expression. Microglia expressing high levels of Iba-1 and astrocytes expressing high levels of GFAP were significantly enhanced in ipsilateral penumbra at 24 h after H/I, indicating that microglia and astrocytes were activated under hypoxic conditions (Supplementary Fig. 2c,d). In addition, TIM-3 expression was meaningfully higher in Iba-1-positive microglial cells and GFAP-positive astrocytes isolated from ipsilateral cortex compared with those from contralateral regions (Fig. 1e,f and Supplementary Fig. 2c,d). These results support our contention that TIM-3 expression is significantly elevated in activated microglia and astrocytes under hypoxia.

### TIM-3 is elevated in a HIF-1-dependent manner under hypoxia

**Figure 2:**
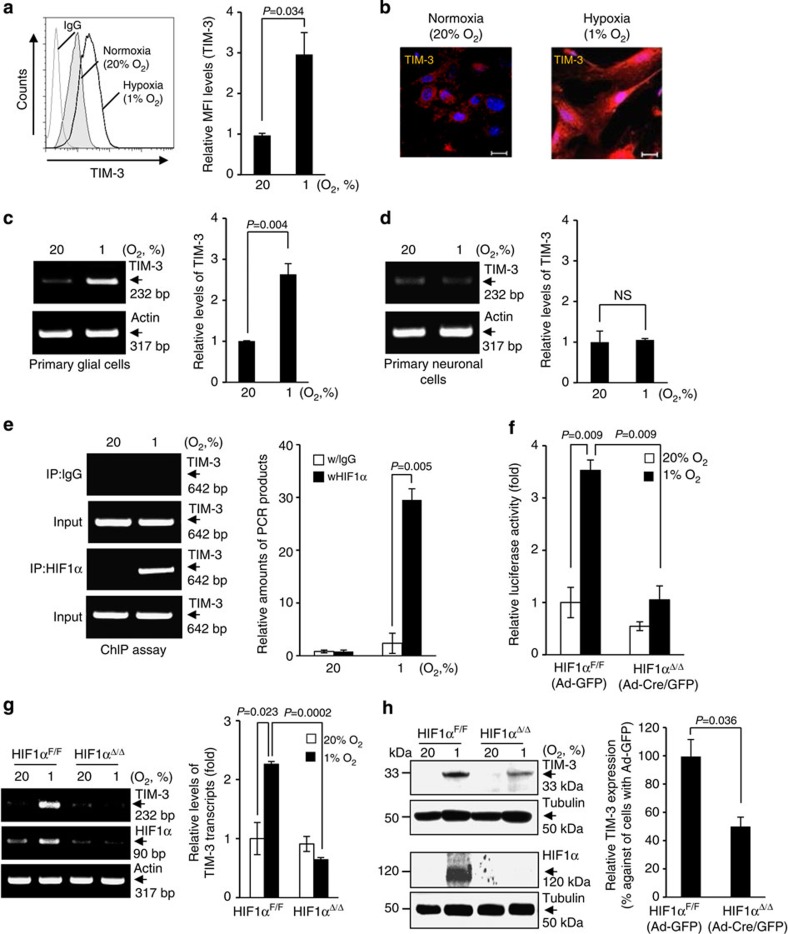
HIF-1α binds to the TIM-3 promoter and regulates its expression in primary glia. (**a**) Cell surface expression of TIM-3 was analysed in BV2 cells under 20% O_2_ or 1% O_2_ for 24 h by flow cytometry using PE-conjugated anti-TIM-3 antibody. The results from three independent experiments are presented as a representative histogram and the mean fold change (± s.d.) relative to the normoxic sample. (**b**) Mouse primary mixed glial cells were incubated under hypoxia or normoxia for 24 h, and the cells were examined by immunocytochemistry using an anti-TIM-3 antibody. (**c**,**d**) Mouse primary mixed glial cells and primary neuronal cells were incubated under hypoxia or normoxia for 24 h, and then RT–PCR was used to detect the levels of TIM-3 and actin. Relative transcript levels are shown as the mean fold change (± s.d.) from three independent experiments (NS, not significant, Student–Newman–Keuls test). (**e**) Primary mixed glial cells were incubated under hypoxia or normoxia for 24 h, and chromatin immunoprecipitation (ChIP) was performed with anti-HIF-1α or control IgG. The results are presented as relative amounts representative of three independent experiments. (**f**) Primary mixed glial cells were cultured from *HIF-1α*^*+f/+f*^ mice, infected with Ad-GFP or Ad-Cre/GFP, transfected with TIM-3-luciferase reporter constructs and incubated under hypoxic or normoxic conditions for 24 h. Relative promoter activity is expressed as the ratio of luciferase activity/β-galactosidase activity. (**g**,**h**) RT–PCR (**g**) and western blot analysis (**h**) were performed under hypoxia or normoxia for 24 h using the indicated primers and antibodies, respectively. The data shown are representative of at least three independent experiments. The graphs show the percent changes in TIM-3 transcript and protein levels in Ad-Cre/GFP- versus Ad-GFP-infected cells under hypoxia. IP, immunoprecipitation.

On the basis of the above observations, we next examined whether TIM-3 expression in glial cells could be altered by oxygen tension, using the BV2 microglial cells and primary cultured glial cells. BV2 cells were incubated under normoxic (20% O_2_) or hypoxic (1% O_2_) conditions for 24 h, and the cell surface levels of TIM-3 were determined by FACS analysis. Interestingly, TIM-3 expression was markedly elevated under hypoxia (Fig. 2a). Immunocytochemistry also demonstrated that TIM-3 expression was considerably higher in mouse primary mixed glial cells under hypoxia versus normoxia (Fig. 2b). In addition, we observed that TIM-3 transcript levels were increased in primary mixed glial cells under hypoxia, but not in primary neuronal cells under the same conditions (Fig. 2c,d and Supplementary Fig. 2e). These results suggest that hypoxia induces TIM-3 expression in glial cells.

HIF-1 is a master transcriptional regulator of numerous genes under hypoxic conditions. To examine whether the hypoxia-stimulated upregulation of TIM-3 was mediated by HIF-1 in glial cells, we performed chromatin immunoprecipitation (ChIP) assays using an anti-HIF-1α antibody and TIM-3 promoter elements containing putative HIF-responsive element (HRE) consensus sequences. As shown in Fig. 2e, HIF-1α was able to bind to the HRE-containing TIM-3 promoter elements in primary mixed glial cells under hypoxia. To further demonstrate this, we examined TIM-3 promoter activity in HIF-1α-deficient glial cells. Primary mixed glial cells were cultured from *HIF-1α*^flox/flox^ (*HIF1α*^*+f/+f*^) mice and then infected with adenovirus-Cre/GFP (Ad-Cre/GFP) or control GFP (adenovirus encoding green fluorescence protein (Ad-GFP)). We confirmed the efficiency of viral infection using FACS, then transfected the cells with a TIM-3 luciferase reporter (−1,517/+1) and measured TIM-3 promoter activity. As expected, TIM-3 promoter activity was significantly enhanced in control Ad-GFP-infected glial cells (HIF1α^F/F^) under hypoxic conditions, but it was markedly reduced in Ad-Cre/GFP-infected, HIF-1α-deficient glial cells (HIF1α^Δ/Δ^; Fig. 2f). Site-directed mutagenesis of the putative HREs in the TIM-3 promoter significantly diminished the hypoxia-dependent increase of luciferase activity compared with the wild-type reporter (Supplementary Fig. 3). In addition, hypoxia-stimulated increase of TIM-3 transcripts and proteins was markedly suppressed in Ad-Cre/GFP-infected HIF-1α-deficient glial cells (Fig. 2g,h). These results support that TIM-3 expression is regulated in a HIF-1-dependent manner under hypoxia.

### Blocking TIM-3 reduces brain damage in the mouse H/I model

**Figure 3:**
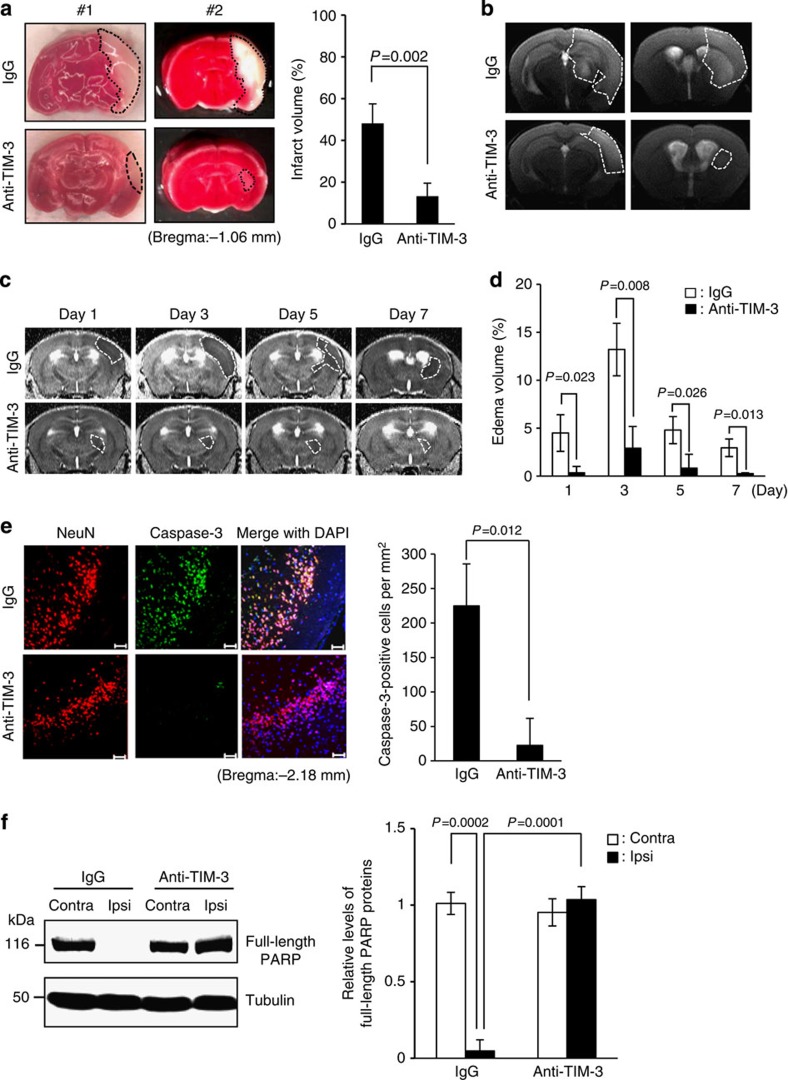
Blocking TIM-3 significantly reduces brain injury after hypoxia-ischaemia. (**a**) Representative images of TTC-stained brain slices from H/I mice treated with 100 μg of IgG (*n*=12) or anti-TIM-3 antibody (*n*=12). The infarct volume was quantified with Image J analyser and expressed as a percentage of the damaged ipsilateral hemisphere. (**b**) Representative magnetic resonance images (MRIs) from TIM-3-antibody-treated mice (*n*=4) and IgG-treated mice (*n*=4) at 24 h post-H/I. (**c**) Representative T2 images from TIM-3-antibody-treated mice (*n*=4) and IgG-treated mice (*n*=4) after H/I. (**d**) The extent of the oedema formation was obtained from the T2-weighted MRI images and ADC map. (**e**) Representative confocal microscopic images of immunohistochemical staining for NeuN and cleaved caspase-3 in coronal brain sections from IgG- and anti-TIM-3-treated H/I mice 24 h after injury. Scale bar, 50 μm. The graph shows the mean number of NeuN and cleaved caspase-3-stained cells per mm^2^. (**f**) Immunoblot detection of full-length PARP proteins in contralateral and ipsilateral cortex regions of control IgG- or anti-TIM-3-treated mice. The graph shows the relative levels of full-length PARP (116 kDa). Data represent the mean±s.d. from at least three independent experiments.

Given that TIM-3 is upregulated in glial cells subjected to H/I, we explored the role of hypoxia-induced TIM-3 in the brain after cerebral H/I. For this, we investigated the effect of a TIM-3-blocking antibody on brain damage 24 h post H/I using 2,3,5-triphenyltetrazolium (TTC) staining. As shown in Fig. 3a, the TTC-negative area was significantly reduced in mice that were given intravenous injections of 100 μg TIM-3-blocking antibody, compared with control IgG-injected mice. These results suggest that the TIM-3-blocking antibody may ameliorate brain damage under hypoxic conditions.

Oedema, a life-threatening consequence of brain infarction, is accompanied by inflammation and the consequent extension of ischaemic brain lesions^40^. We thus assessed the effect of TIM-3 blockade on the formation of oedema following H/I. To monitor infarct area and oedema formation, we acquired T2-weighted magnetic resonance images from days 1 to 7 post-H/I. Similar to the results obtained from TTC staining, the infarction and formation of oedema in the ipsilateral hemispheres of TIM-3-antibody-injected mice was significantly reduced compared with that in IgG-injected mice on day 1 post-H/I (Fig. 3b–d). These decreases in oedema formation and infarction persisted on days 3, 5 and 7 days post insult (Fig. 3c,d and Supplementary Fig. 4a–c).

To further investigate the involvement of TIM-3 in post-H/I brain damage, we examined the effect of the TIM-3-blocking antibody on neuronal cell death by assessing the expression of caspase-3, a key cell death effector protease that plays a crucial role in cerebral ischaemia^41^,^42^. Immunohistochemistry showed that the expression of caspase-3 in neuronal cells was significantly elevated in the ipsilateral cortex regions of IgG-treated H/I mice, whereas this elevation was dramatically diminished in mice treated with TIM-3 blocking antibody (Fig. 3e). We next examined the level of poly (ADP-ribose) polymerase (PARP), a marker for caspase-3 activity that is cleaved by caspase-3 and has been implicated in ischaemic cell death^41^,^43^, in the ipsilateral and contralateral cortex of H/I mice treated with control IgG or the TIM-3-blocking antibody. As shown in Fig. 3f, the expression of full-length PARP was markedly decreased in tissues from the ipsilateral cortex of control IgG-injected H/I mice, but not TIM-3-blocking antibody-injected H/I mice. These findings show that blocking of TIM-3 may significantly reduce infarct volume and neuronal cell death after cerebral ischaemia in mice.

### Neutrophil infiltration is attenuated by blocking TIM-3

Studies have shown that neutrophils are rapidly infiltrated into the ischaemic brain within hours, contributing to inflammatory events and brain damage^44^,^45^. Given that glial cells are among the first cells that respond to brain injury, exhibiting relevant activities within just minutes of ischaemia onset^5^, we hypothesized that a HIF-1-dependent increase of TIM-3 in glial cells would influence the infiltration of neutrophils into ischaemic penumbra, and speculated that downregulation of the ability of TIM-3 to recruit neutrophils could reduce brain damage after cerebral ischaemia. We first assessed the expression of myeloperoxidase (MPO) and granulocyte receptor-1 (Gr-1), two representative neutrophil markers, and confirmed that cells positive for these markers (MPO^+^Gr-1^+^) were markedly increased in the penumbral cortex and striatum compared with contralateral regions at 24 h after H/I (Supplementary Fig. 5). Next, we examined whether glial cells could recruit Gr-1^high^CD11b^high^ neutrophils under hypoxic conditions. Splenocytes were isolated from C57BL/6 mice and incubated in a Transwell system with or without primary mixed glial cells or murine embryonic fibroblast control cells that reportedly recruit immune cells to injured sites^46^ for 24 h under 1 or 20% O_2_ conditions. In the presence of glial cells or murine embryonic fibroblasts, Gr-1^high^CD11b^high^ cells markedly migrated to the lower chamber under hypoxic conditions, whereas only a few cells migrated under normoxic conditions (Supplementary Fig. 6a). However, this hypoxia-dependent increase of migration of Gr-1^high^CD11b^high^ cells was significantly attenuated in the absence of glial cells (Supplementary Fig. 6b). These results suggest that glial cells may be involved in recruiting Gr-1^high^CD11b^high^ cells under hypoxia.

**Figure 4:**
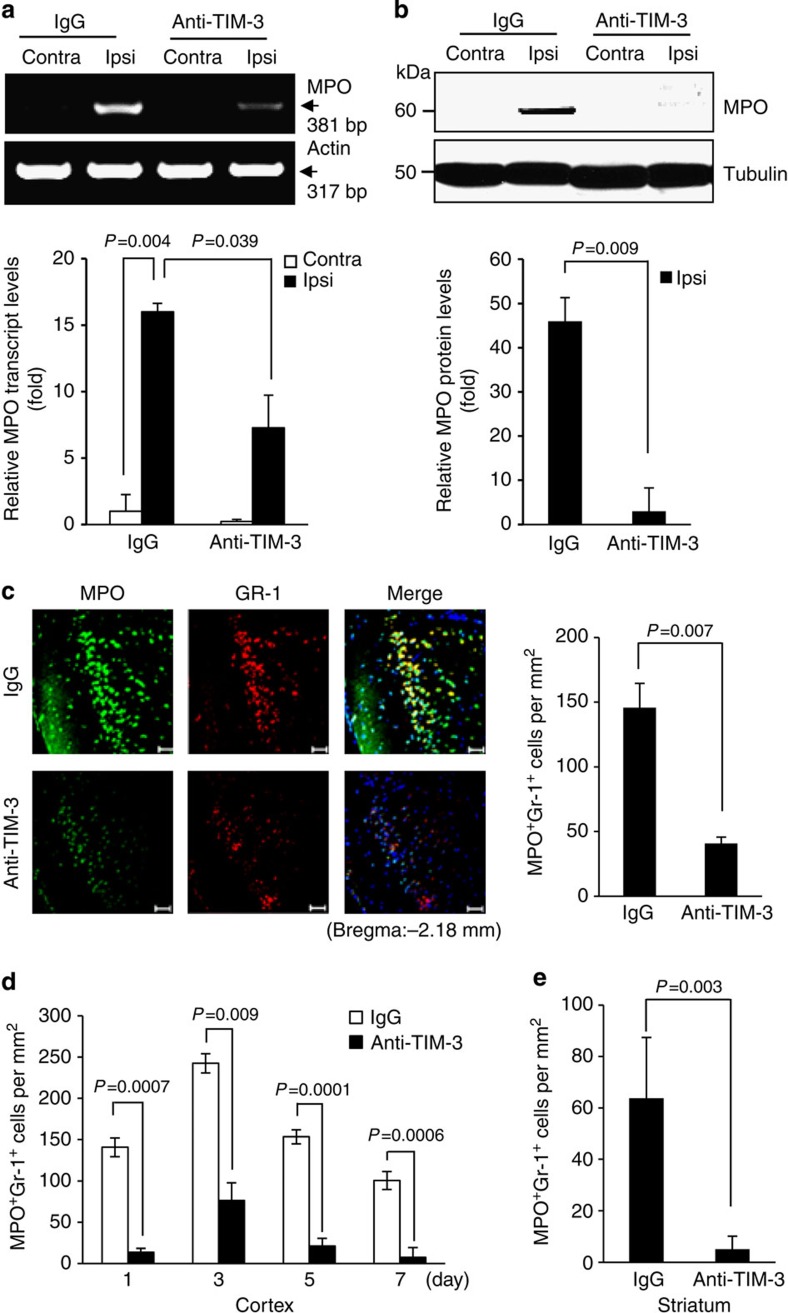
The TIM-3-blocking antibody reduces neutrophil migration. RT–PCR (**a**) and western blot analyses (**b**) were used to examine MPO expression levels in the cortex tissues from control IgG- or anti-TIM-3-treated H/I mice. Graphs show the relative levels of MPO expression. (**c**) Immunohistochemistry was performed on coronal sections from control IgG- or TIM-3-blocking-antibody-treated H/I mice 24 h after injury using antibodies against MPO and Gr-1. Scale bar, 50 μm. The graph shows the mean number of MPO^+^Gr-1^+^ cells per mm^2^ (± s.d.). (**d**) Cortical and (**e**) striatum penumbral regions of coronal sections from the H/I mice were subjected to immunohistochemistry using an anti-MPO and anti-Gr-1 antibodies, and MPO- and Gr-1-positive cells per mm^2^ were counted.

Next, we examined the effect of TIM-3-blockade on neutrophil infiltration into ipsilateral hemispheres at 24 h after H/I. Reverse transcription–PCR (RT–PCR) and western blot analyses of cortex tissues from the H/I mice showed that MPO expression levels were significantly reduced in TIM-3-blocking antibody-treated mice compared with control IgG-treated mice (Fig. 4a,b). Immunohistochemistry of coronal sections from ipsilateral cortex also showed that MPO^+^Gr-1^+^ cells were significantly reduced by TIM-3-blocking antibody treatment (Fig. 4c). These results were confirmed by immunohistochemistry using anti-neutrophil and anti-MPO antibodies (Supplementary Fig. 7). Using coronal sections from several ipsilateral regions of the H/I brain (bregma −2 to +2), we further examined the effect of TIM-3 blockade on neutrophil infiltration at various time points. As shown in Fig. 4d,e and Supplementary Fig. 8a–d, fewer MPO^+^Gr-1^+^ cells were observed in the penumbral cortex and striatum of mice subjected to TIM-3 blockade at all tested time points (days 1–7). Collectively, these findings strongly suggest that TIM-3 may be associated with the infiltration of neutrophils into the injured brain under hypoxic conditions.

### TIM-3 blockade reduces recruitment of neutrophils by glia

**Figure 5:**
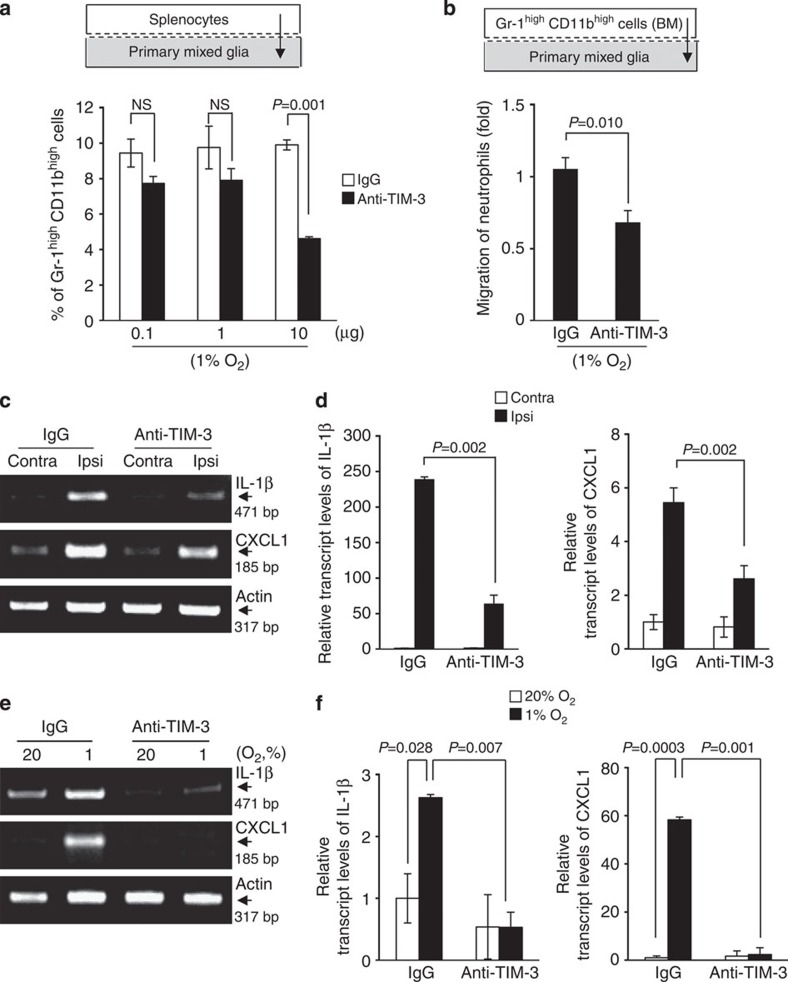
The expression levels of two representative neutrophil chemoattractants are diminished by blocking TIM-3 *in vitro* and *in vivo.* (**a**) Mouse primary mixed glial cells (2 × 10^5^) were plated into the lower chamber of the Transwell system, and pretreated with the indicated concentration of TIM-3-blocking antibody or control IgG, after which 5 × 10^5^ splenocytes were plated into the upper chamber. After incubation for 24 h under hypoxia, the splenocytes that had migrated to the lower chamber were analysed by flow cytometry. The percentage of Gr-1^high^CD11b^high^ cells is expressed as the mean±s.d. from three independent experiments. (**b**) Gr-1^high^CD11b^high^ neutrophils were sorted from the bone marrow of C57BL/6 mice, and incubated with control IgG- or anti-TIM-3-treated primary glial cells under 1% O_2_. The results from three independent experiments are expressed relative to the results from control IgG-treated primary glial cells, which were arbitrarily set to 1. (**c**) RT–PCR analysis was performed on tissue-specific samples from control IgG- or TIM-3-blocking antibody-treated H/I mice. (**d**) Graphs show quantified values normalized to those of actin (*n*=3). (**e**) Primary cultured glial cells were treated with control IgG or TIM-3-blocking antibody, and then incubated under 1 or 20% O_2_ for 24 h. The transcript levels of IL-1β and CXCL1 were determined by RT–PCR analysis. (**f**) The graphs show the results from at least three independent experiments. NS, not significant.

To more specifically assess the influence of glial TIM-3 on neutrophil migration, we examined whether the blocking TIM-3 affected the ability of glia to recruit neutrophils under hypoxia. Using a Transwell system, we plated primary glial cells into the lower chamber, pretreated these cells with the TIM-3-blocking antibody or control IgG, and then loaded the upper chamber with splenocytes. The cells were incubated under 1% O_2_ for 24 h, and the proportion of Gr-1^high^CD11b^high^ cells in the lower chamber was determined by FACS analysis. Our results revealed that Gr-1^high^CD11b^high^ cells in the lower chamber under hypoxia were considerably reduced in the presence of 10 μg of TIM-3-blocking antibody, compared with control IgG (Fig. 5a).

To further validate the above results, we examined the migration of bone marrow (BM)-derived Gr-1^high^CD11b^high^ cells under hypoxic conditions. Gr-1^high^CD11b^high^ cells were sorted from BM cells, plated into the upper chamber, and the lower chamber was loaded with TIM-3-blocking antibody- or control IgG-treated primary mixed glial cells under 1% O_2_ (Supplementary Fig. 6c). Consistent with the results described above, the migration of BM-derived Gr-1^high^CD11b^high^ cells to the lower chamber was significantly reduced by TIM-3-blocking antibody treatment compared with control IgG treatment (Fig. 5b). These results clearly support the role for glial TIM-3 in recruiting neutrophils to hypoxic regions after cerebral ischaemia.

### TIM-3 blockade decreases neutrophil chemoattractants

The infiltration of neutrophils into inflamed or injured sites is regulated by chemoattractants, which are upregulated before neutrophil infiltration of the brain following ischaemia^34^. We thus examined the effect of the TIM-3 blockade on the levels of IL-1β and CXCL1, which act as neutrophil chemoattractants in the ischaemic brain^44^,^47^. Mice were intravenously injected with 100 μg of TIM-3-blocking antibody or control IgG at 30 min after H/I. Twenty-four hours later, the transcript levels of IL-1β and CXCL1 were examined in tissues from ipsilateral and contralateral cortex. As shown in Fig. 5c,d, the levels of both IL-1β and CXCL1 transcripts were markedly elevated in ipsilateral cortex regions of H/I mice injected with control IgG, but this effect was significantly reduced in mice injected with TIM-3-blocking antibody.

To further assess the role of glial TIM-3, we investigated the effect of TIM-3 blockade on the expression levels of IL-1β and CXCL1 in primary glial cells. The cells were treated with the TIM-3-blocking antibody or control IgG, and incubated under 1% O_2_ or 20% O_2_ for 24 h. Consistent with the above results, the levels of IL-1β and CXCL1 transcripts were considerably increased in IgG-treated control cells incubated under 1% O_2_ compared with 20% O_2_, but these enhancements were markedly reduced in cells treated with the TIM-3-blocking antibody (Fig. 5e,f). These results further support our hypothesis that glial TIM-3 may be an important player in the pathogenesis of cerebral ischaemia via the regulation of neutrophil infiltration.

### HIF-1 deficiency reduces neutrophil migration and infarct

**Figure 6:**
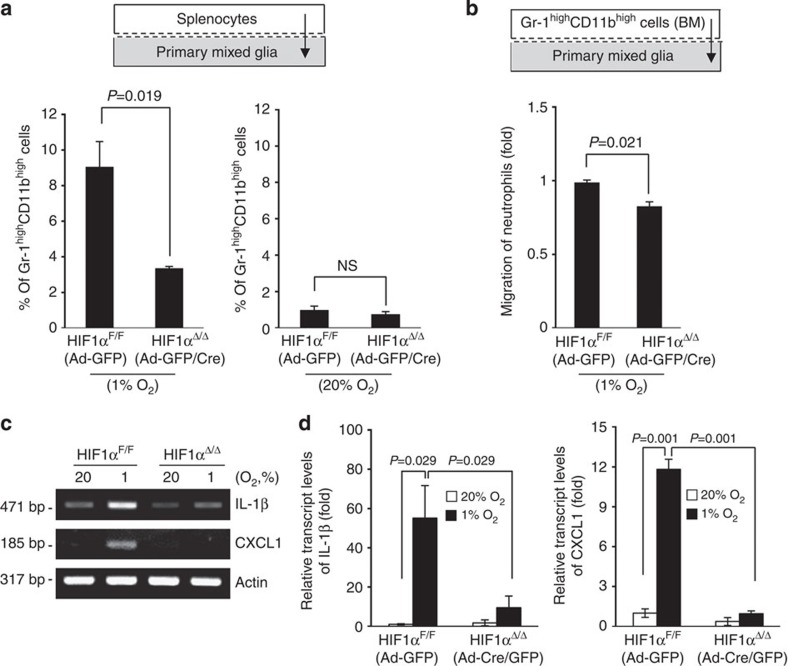
The hypoxia-triggered migration of neutrophils is reduced under HIF-1α-deficiency. (**a**) Primary mixed glial cells (2 × 10^5^) from *HIF-1α*^*+f/+f*^ mice were infected with Ad-GFP or Ad-Cre/GFP, and plated to the lower chamber of the Transwell apparatus, and 5 × 10^5^ splenocytes were plated to the upper chamber. After incubation for 24 h under hypoxia, the cells that had migrated into the lower chamber were analysed by flow cytometry. (**b**) Gr-1^high^CD11b^high^ were sorted from the bone marrow of C57BL/6 mice and incubated in a Transwell system along with Ad-GFP or Ad-Cre/GFP-infected primary mixed glial cells under hypoxia. The results from three independent experiments are presented relative to those from Ad-GFP-infected HIF-1α^F/F^ glial cells. (**c**) The transcript levels of IL-1β and CXCL1 were determined in primary glia infected with Ad-GFP or Ad-Cre/GFP and cultured under 1% or 20% O_2_ for 24 h. (**d**) The graphs show the results from real-time quantitative PCR.

Given our observation that HIF-1α controls expression of TIM-3 in glial cells under hypoxia, we examined whether HIF-1α could influence the ability of glial cells to recruit neutrophils under hypoxic conditions. Primary mixed glial cells cultured from *HIF-1α*^*+f/+f*^ mice were infected with Ad-GFP or Ad-GFP/Cre, and incubated in a Transwell system with splenocytes under 1% O_2_ or 20% O_2_ for 24 h. The proportion of Gr-1^high^ CD11b^high^ cells in the lower chamber under hypoxia was significantly decreased when splenocytes were incubated with Ad-GFP/Cre-infected HIF-1α-deficient glial cells, compared with control Ad-GFP-infected cells. In contrast, the number of migrated Gr-1^high^ CD11b^high^ cells did not significantly differ between HIF-1α-deficient and normal cells under 20% O_2_ (Fig. 6a). We next found that the number of migrated BM-derived Gr-1^high^CD11b^high^ cells was meaningfully reduced following incubation with HIF-1α-deficient glial cells under 1% O_2_ (Fig. 6b). In addition, the hypoxia-dependent increases of IL-1β and CXCL1 were significantly reduced in Ad-GFP/Cre-infected HIF-1α-deficient glial cells, where hypoxia-dependent increase of TIM-3 was not detected, compared with control Ad-GFP-infected cells (Fig. 6c,d)

**Figure 7:**
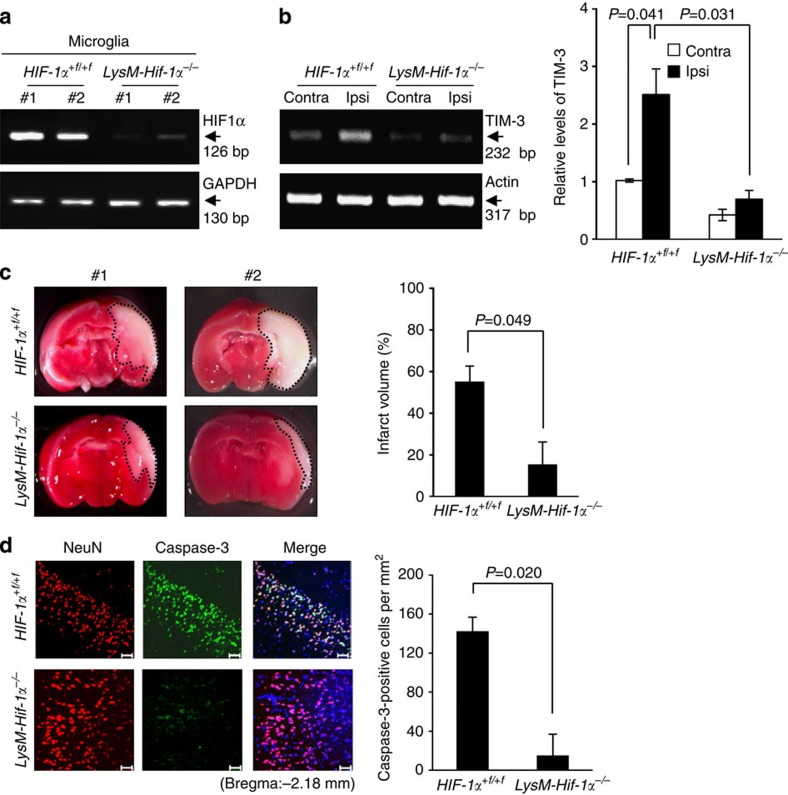
H/I-induced brain damage is reduced in *LysM-Hif-1α*^−*/*−^ mice. (**a**) RT–PCR analysis was performed in primary microglia cultured from *HIF-1α*^*+f/+f*^ or *LysM-Hif-1α*^−*/*−^ mice using the indicated primers. (**b**) TIM-3 transcript levels were examined in bran tissues from the contralateral cortex and ischaemic ipsilateral cortex of *HIF-1α*^*+f/+f*^ or *LysM-Hif-1α*^−*/*−^ mice (*n*=3) at 24 h post-H/I. (**c**) Representative images of TTC-stained brain slices from in H/I-induced *HIF-1α*^*+f/+f*^ (*n*=12) or *LysM-Hif-1α*^−*/*−^ (*n*=12) mice 24 h after injury. The infarct volume was quantified with Image J analyser and expressed as a percentage of the damaged ipsilateral hemisphere. (**d**) Representative confocal microscopic images of immunohistochemical staining for NeuN and cleaved caspase-3 in coronal brain sections from H/I-induced *HIF-1α*^*+f/+f*^ or *LysM-Hif-1α*^−*/*−^ mice 24 h after injury. Scale bar, 50 μm. The graph shows the mean number of NeuN and cleaved caspase-3-stained cells per mm^2^ (±s.d. from three independent experiments).

Microglia have been reported to be resident myeloid cells in the brain^48^. In an attempt to ascertain the role of glial HIF-1α, we investigated the extent of brain damage after H/I in *LysMCre-HIF-1α*^*+f/+f*^ (*LysM-Hif-1α*^−*/*−^) mice, which lack HIF-1α specifically in myeloid cells. We first examined the level of HIF-1α in primary microglia from *LysM-Hif-1α*^−*/*−^ mice. As shown in Fig. 7a and Supplementary Fig. 9a, HIF-1α transcript levels were markedly lower in microglia from *LysM-Hif-1α*^−*/*−^ mice compared with *HIF-1α*^*+f/+f*^ mice. TIM-3 transcript levels were also lower in the ipsilateral cortex regions of *LysM-Hif-1α*^−*/*−^ mice at 24 h post H/I (Fig. 7b). We observed that the TTC-staining-negative area was markedly reduced in *LysM-Hif-1α*^−*/*−^ mice compared with *HIF-1α*^*+f/+f*^ mice, indicating a role for microglial HIF-1α in brain damage 24 h after H/I (Fig. 7c). Expression of caspase-3 in neuronal cells was also meaningfully decreased *LysM-Hif-1α*^−*/*−^ mice compared with *HIF-1α*^*+f/+f*^ mice (Fig. 7d). Furthermore, we failed to detect any significant increase of IL-1β and CXCL1 expression in the ipsilateral cortex of *LysM-Hif-1α*^−*/*−^ mice at 24 h post H/I (Supplementary Fig. 9b). These results imply that HIF-1α may be closely involved in TIM-3-associated neutrophil infiltration and subsequent brain damage under hypoxia.

### Both TIM-3 blockade and HIF-1α deficiency influence NDS

To determine whether the reduced infarct volume and neuronal cell death are correlated with improvement in neurological function, we measured the neurological deficit score (NDS) in the H/I model using widely used methods^49^,^50^. The neurological deficits were assessed by flexion of contralateral torso and forelimb, circling to the contralateral side, leaning to the contralateral side at rest, and spontaneous motor activity. Neurological deficits caused by H/I were decreased in mice treated with TIM-3-blocking antibody compared with IgG-treated mice. Twenty hours after H/I, the NDS for mice treated with IgG was 2.8±0.8 (±s.d.), whereas the NDS for mice treated with TIM-3-blocking antibody was 0.8±0.8 (Table 1a; *P*=0.012; Mann–Whitney *U*-test). These results suggest that TIM-3 is associated with neurological function under hypoxia. Next, we assessed the NDS in *HIF-1α*^*+f/+f*^ mice (*n*=10) and *LysM-Hif-1α*^−*/*−^ mice (*n*=11) at 24 h after H/I. Leaning behaviour and an absence of spontaneous motor function were observed in *HIF-1α*^*+f/+f*^ mice, but not in *LysM-Hif-1α*^−*/*−^ mice (Supplementary Movies 1–4). The average NDS in *LysM-Hif-1α*^−*/*−^ mice were significantly lower than that in *HIF-1α*^*+f/+f*^ mice (Table 1b; 1.2±0.6 versus 2.6±1.1, *P*=0.0008). Taken together, these findings suggest that the HIF-1α/TIM-3 axis may be closely involved in neurological function as well as cerebral infarct volume and pathophysiological inflammatory events.

### TIM-3 increases neuronal damage in HIF-1α-deficient mice

**Figure 8:**
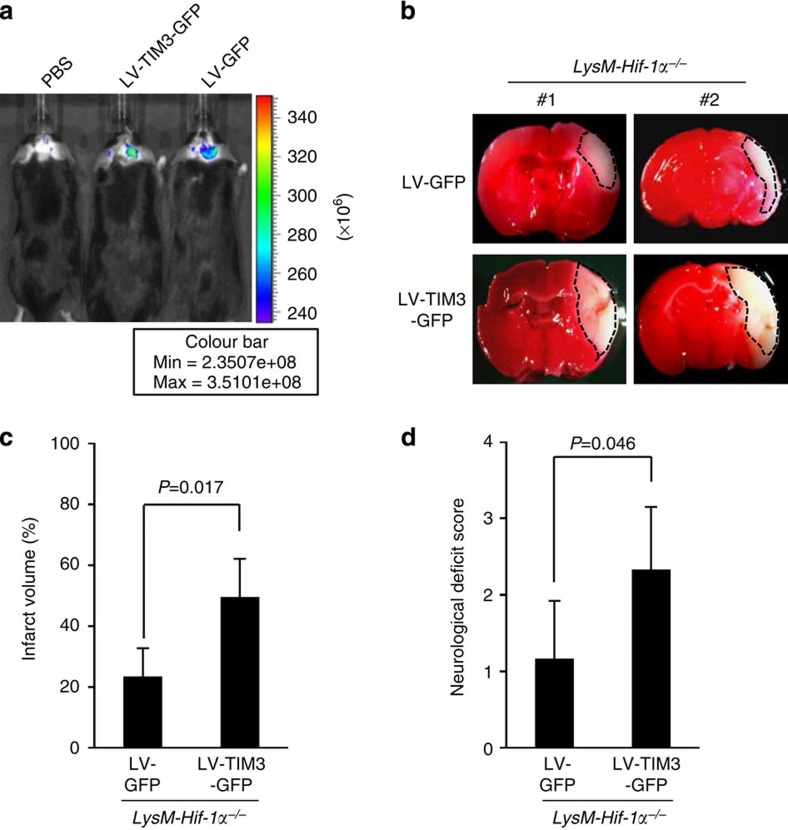
Intracranial injection of LV-TIM3-GFP into the brain of *LysM-Hif-1α*^−*/*−^ mice increases infarct size and neurological deficit. (**a**) Representative fluorescence images (excitation filter, from 445 to 490 nm, and emission filter, from 515 to 575 nm) of mice injected with PBS, LV-GFP or LV-TIM3-GFP using IVIS Spectrum system (Xenogen IVIS-200). (**b**) Representative images of TTC-stained brain slices from mice expressing LV-TIM3-GFP or LV-GFP. (**c**,**d**) Infarct size (**c**, *n*=6 for LV-GFP or *n*=5 for LV-TIM3-GFP) and neurological score (**d**, *n*=6 for each group) were examined 24 h after H/I. The data were analysed by Mann–Whitney *U-*test; *P*=0.046.

We next examined whether TIM-3 could influence the phenotype of HIF-1α-deficient mice after H/I. For this, we generated a lentiviral vector expressing TIM-3 and GFP (LV-TIM3-GFP). We first examined whether the lentiviruses were capable of infecting glial cells and observed that TIM-3 expression was significantly increased in GFP-positive-CD11b^high^CD45^low^ glial cells from lentivirus-injected mice (Supplementary Fig. 10a). We injected the viruses into the right hemisphere of *LysM-HIF-1α*^−*/*−^ mice using a stereotaxic instrument. Control mice were injected with LV-GFP, expressing GFP alone. Each mouse was subjected to four intracranial injections in the right hemisphere (Fig. 8a and Supplementary Fig. 10b). H/I was induced 5 days after injection of *LysM-Hif-1α*^−*/*−^ mice with LV-TIM3-GFP or LV-GFP, and infarct size and neurological outcomes were examined 24 h later. As shown in Fig. 8b,c, the TTC-staining-negative area was significantly increased in mice given injections of LV-TIM3-GFP (*n*=5), compared with control LV-GFP-injected mice (*n*=6). In addition, the average NDS for *LysM-Hif-1α*^−*/*−^ mice injecting LV-TIM3-GFP were higher than that for LV-GFP-injected control mice (Fig. 8d and Supplementary Fig. 10C) (1.1±0.7 versus 2.3±0.8, *P*=0.046). These results further support the involvement of the HIF-1/TIM-3 axis in brain injury under hypoxia.

## Discussion

Clinical and experimental findings indicate that inflammatory processes are critically involved in the pathogenesis of ischaemic brain injury, and that patients with abnormally elevated inflammation exhibit poorer outcomes^5^,^44^. In addition, strategies aimed at limiting pathological inflammatory processes have been shown to have therapeutic potential in experimental models of brain ischaemia. Thus, researchers have recently focused on developing anti-inflammatory agents that target immune and inflammatory cells/mediators for the treatment of brain ischaemia^47^,^51^. In this study, we explored molecular players that could play pivotal roles in the interdependent associations between post-ischaemic inflammatory events and brain damage.

Tissues in the ischaemic core are irreversibly damaged, but penumbral tissues are metastable and potentially salvageable. The penumbra includes ischaemic area that recover spontaneously and areas that progress to irreversible changes unless effectively treated^2^,^52^. Thus, salvageable tissue is the potential target for therapeutic intervention. Our experiments in a H/I mouse model and a primary glial cell culture system revealed that TIM-3 expression is elevated under hypoxic conditions, predominantly in activated microglia and astrocytes of penumbral regions, where it may play a crucial role in inflammatory processes associated with neutrophil infiltration. Accumulating evidence has shown that, under pathological conditions, expression of TIM-3 can be induced in some cells, where it appears to play multiple roles in both adaptive and innate immunity^7^. In this context, we hypothesized that hypoxia-induced glial TIM-3 expression could be involved in pathophysiological immune responses following cerebral ischaemia, and tested this by examining the characteristic features and functions of TIM-3 in post-ischaemic responses.

HIF-1 is generally accepted to be a pivotal physiological regulator of anaerobic metabolism, as well as an essential modulator of immunological responses^22^,^25^,^28^. In addition, HIF-1 has been suggested to play an essential role in hypoxic-ischaemic brain damage, and has been proposed as therapeutic target for the treatment of ischaemic diseases^53^. Given our observation that TIM-3 expression is regulated by oxygen tension, we examined whether HIF-1 was capable of modulating TIM-3 expression in the activated glial cells of H/I mice. ChIP and promoter activity assays showed that HIF-1 bound to the TIM-3 promoter and regulated its activity under 1% O_2_. Experiments using HIF-1α-deficient glial cells strongly support that HIF-1 might critically modulate glial TIM-3 expression under brain hypoxic conditions. These results collectively suggest that TIM-3 serves as a downstream mediator of HIF-1 in the inflammatory processes associated with hypoxic-ischaemic damage.

To examine whether TIM-3 could indeed contribute to the pathogenesis of brain ischaemia, we investigated the influence of TIM-3 upregulation on the brain damage and pathological inflammatory responses observed following H/I. Notably, we found that blocking TIM-3 using a monoclonal antibody significantly reduced infarct size and oedema formation compared with that in IgG-treated control H/I mice. Previous reports have shown that TIM-3 is associated with several CNS diseases. For example, TIM-3 has been found to play important roles in inducing and determining the severity of EAE^6^,^17^. Recently, Zhao *et al.* reported that TIM-3 expression is augmented in peripheral blood mononuclear cells of ischaemic stroke patients and brain tissues of global ischaemia-reperfusion mice, suggesting a possible role for TIM-3 in brain ischaemic disease^54^. Our findings, taken together with these previous reports, suggest that TIM-3 may be closely associated with the pathogenesis of inflammation-related CNS diseases.

TIM-3 has been implicated in diverse pathophysiological events, including both activation and inhibition of immune responses, and induce distinct signalling responses in various cell types and conditions^8^,^55^. TIM-3 blockade was shown to increase Th1 inflammation and trigger immune-mediated tissue injury in autoimmune diseases and transplant rejection models^56^. In contrast, activation of TIM-3 on antigen-presenting cells was shown to play a proinflammatory role and contribute to the regulation of inflammation-associated diseases^8^. Notably, the outcome of TIM-3 signalling may differ even within the same cell type in a context-dependent manner. For example, TIM-3 has been reported to suppress nucleic acid-mediated innate immune responses in tumour-infiltrating dendritic cells (DCs)^9^, whereas TIM-3 promotes lipopolysaccharide-induced DC activation^8^. Considering that these reports indicate that TIM-3 has multiple functions, we sought to carefully decipher the role of glial TIM-3 and the outcome of TIM-3 signalling in our H/I model.

Studies have shown that inflammatory cells infiltrate the brain during brain ischaemia and that this infiltration is closely correlated with the inflammatory status and severity of tissue damage. Blood-derived macrophages are recruited into the ischaemic brain beginning 24–48 h after focal ischaemia, with the most abundant recruitment seen on days 3–7 after stroke^57^,^58^,^59^,^60^,^61^. T cells are reported to infiltrate around the infarct by day 3, and their numbers increase progressively between days 3 and 7 (refs 5, 62, 63). Infiltration of neutrophils into the ischaemic brain begins within 30 min to a few hours of focal cerebral ischaemia, peaks at days 1–3 and disappears thereafter. These infiltrating neutrophils release various cytokines and chemokines, and their massive infiltration exacerbates brain injury^5^. Thus, the balanced regulation of neutrophil infiltration might be an important factor in determining secondary damage after cerebral ischaemia. Brain resident glial cells are among the first cells to respond to brain injury and release various inflammatory mediators. We therefore questioned whether HIF-1-dependent upregulation of TIM-3 on glial cells influenced the infiltration of neutrophils into ischaemic regions under hypoxia. Interestingly, we found that migration of Gr-1^high^CD11b^high^ cells was increased in the presence of glial cells under 1% O_2_. In addition, blockade of TIM-3 significantly reduced the ability of glial cells to recruit neutrophils *in vivo* and *in vitro*, and diminished the levels of IL-1β and CXCL1, which are known to act as neutrophil chemoattractants in the ischaemic brain^44^,^47^. Furthermore, blockade of TIM-3 improved functional neurological deficits in the H/I mouse model. These results indicate that glial TIM-3 may be closely involved in neutrophil infiltration and production of neutrophil chemoattractants in ischaemic brain regions, supporting our hypothesis.

**Figure 9:**
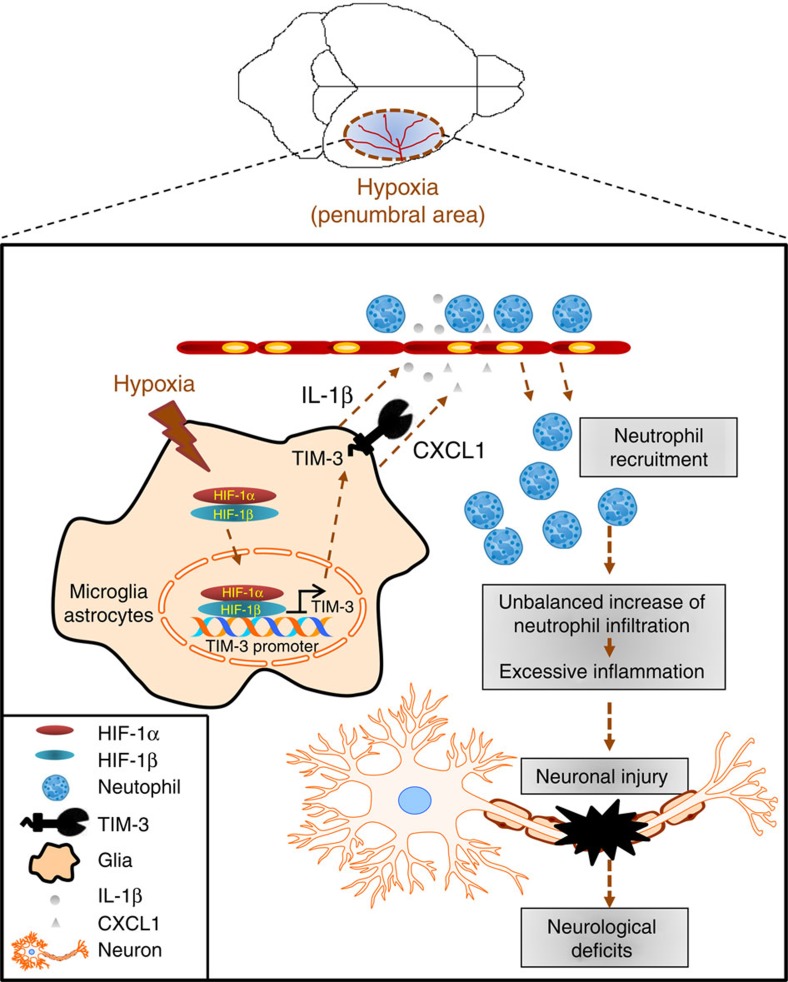
Schematic diagram depicting possible TIM-3-associated events in the brain under hypoxia. The hypoxia-dependent activation of HIF-1 enhances TIM-3 expression in microglia and astrocytes. Activation of the HIF-1/TIM-3 axis induces the production of neutrophil attractants and the recruitment of neutrophils to hypoxic sites. Aberrant increase of neutrophil infiltration causes abnormal inflammation and subsequent pathological events in the brain.

Experiments in HIF-1α-deficient glial cells further support our hypothesis. To more address the question on the HIF-1/TIM-3 axis in hypoxia-associated brain inflammation, we examined neutrophil infiltration in Ad-GFP- or Ad-GFP/Cre-infected glial cells from *HIF-1α*^*+f/+f*^ mice. Consistent with the results obtained with our TIM-3-blocking antibody, both neutrophil infiltration and IL-1β production were significantly diminished in studies using HIF-1α-deficient glial cells. Neurological deficits caused by H/I were also decreased in *LysM-HIf-1α*^−*/*−^ mice lacking microglial HIF-1α. Moreover, intracranial injection of LV-TIM3-GFP into cortical region of *LysM-HIf-1α*^−*/*−^ mice increased infarct area and worsened neurological outcomes. Previously, our group reported that neutrophil infiltration was downregulated in the skins of HIF-1α-null mice subjected to chemical irritation owing to impairment of HIF-1α-regulated metabolic responses^25^. It has also been shown that HIF-1α is directly involved in regulating neutrophil survival under hypoxia^63^. Our current findings provide that TIM-3 is induced by HIF-1α in glial cells under hypoxia, and that this appears to affect the recruitment of neutrophils into ischaemic regions, at least in part through increased production of neutrophil chemoattractants (Fig. 9). Taken together, these results suggest that HIF-1α may affect neutrophil infiltration into the hypoxic brain by regulating the expression of glial TIM-3 as well as that of genes associated with metabolic responses. They also strongly support that infiltration of inflammatory cells is an important factor in brain damage after cerebral ischaemia.

In summary, we here present a function for TIM-3 as a molecular player that links inflammation and brain damage after cerebral ischaemia. We reveal that glial TIM-3 is increased in a HIF-1-dependent manner under hypoxia, and that either TIM-3 blockade or HIF-1α deficiency significantly reduces the ischaemic infarct volume and functional neurological deficits in a H/I mouse model. Further understanding of the function and characteristics of TIM-3 and HIF-1 during cerebral ischaemia, particularly in the balanced regulation of neutrophil infiltration, may contribute to the future development of effective therapeutic approaches against hypoxia-associated brain diseases.

## Methods

### Mice

Mice carrying HIF-1α-floxed alleles (*HIF-1α*^*+f/+f*^) were produced by Dr Randall Johnson, and were maintained in the animal facility at the National Cancer Center (AAALAC accredited facility). Mice lacking HIF-1α in myeloid lineage cells were generated by cross-breeding *HIF-1α*^*+f/+f*^ mice to *LysM-Cre* transgenic mice^25^. Eight0week-old male C57BL/6 mice (Orient Bio) were used for *in vivo* and *in vitro* experiments. All animal procedures were performed according to ARRIVE guidelines and National Cancer Center guidelines for the care and use of laboratory animals. The protocol was approved by the Committee on the Ethics of Animal Experiments of the National Cancer Center (Permit Number: NCC-11-125). To avoid bias, the animal studies in this study were properly randomized in a blinded manner with respect to the genotypes and treatments.

### Hypoxic cerebral ischaemia model and assessment of infarct volume

H/I was induced in C57BL/6 male mice (8 weeks, Orient Bio) as described by Zhang *et al.*^35^ Briefly, mice were anaesthetized with Zoletil (Virbac) and Rompun (Bayer) (4:1), and each mouse’s right common carotid artery was exposed and double-ligated with 4-0 surgical silk. The incisions were sutured, and mice were allowed to recover for 2 h with access to food and water. Systemic hypoxia was induced by exposure to 8% O_2_/balance N_2_ in temperature-controlled hypoxia chambers (BioSpherix, C-474). This model of transient unilateral cerebral ischaemia has been shown to generate reproducible brain damage in the ipsilateral hemisphere, but not in the contralateral hemisphere. For TIM-3-blocking experiments, mice were intravenously injected with 100 μg of rat IgG2a, k isotype (eBioscience, 16-4321) or an anti-TIM-3 monoclonal antibody (eBioscience, RMT-3-23) 30 min after H/I. At 24 h after H/I, mice were killed, and brains were removed and immediately sliced into 2-mm-thick sections, which were incubated with TTC at 37 °C for 30 min. Images of these sections were obtained under stereomicroscope fitted with a camera (Zeiss, Stereo Discovery.V20). Infarct volume, which was determined using an indirect method that compensates for oedema of the infarcted tissue, was calculated as the percentage of the ratio of the damaged area to the area of the hemisphere with correction for hemispheric swelling due to oedema, using the formula: Infarct volume (%)=[(contralateral hemisphere-healthy area in ipsilateral hemisphere)/contralateral hemisphere] × 100 (ref. 64).

### Magnetic resonance imaging assessments

Mice were fixed in an animal bed and placed in an MRI spectrometer (Bruker7T BioSpec), and then anaesthetized during imaging to minimize discomfort. T2-weighted images were acquired using Rapid Acquisition with Relaxation Enhancement sequence. Eighteen contiguous axial slices with a thickness of 0.7 mm were acquired matrix 256 × 256, field of view=20 × 20 mm, TR (Repetition Time)=2,500 ms, TE (Echo Time)=35 ms, acquisition time=4 min and no gap. A map of apparent diffusion coefficient (ADC) was obtained by diffusion-weighted images using a spin-echo sequence. For this, eight contiguous axial images were acquired (thickness 0.7 mm, matrix 256 × 128, field of view=20 × 20 mm, TR=2,000 ms, TE=26.936 ms, acquisition time=16 min, 1 average, *b* values=45, 350, 1,000 and 2,000 s per mm^2^ and no gap). The ADC maps were obtained from scanner. Oedema volumes were calculated with the T2-weighted images and the ADC maps by the Image J analyser. Oedema volume (%)=[(Ipsilateral volume—contralateral volume)/contralateral volume] × 100.

### Isolation of microglia and astrocytes from mouse brain tissues

Microglia were isolated from brain tissue using the previously described Percoll-gradient isolation technique^65^. Briefly, brains were removed from perfused mice, divided into ipsilateral and contralateral hemispheres, minced and digested by incubation with 250 μg ml^−1^ collagenase IV/DNase I at 37 °C for 45 min each. The resulting cell suspensions were fractionated on 50/70% Percoll gradients at 1,000 *g* for 25 min. Microglial cells were collected from the interface between the 50 and 70% bands and washed with hanks' balanced salt solutions (HBSS, Welgene). The purity of the isolated microglial cells was determined by FACS analysis. Astrocytes were isolated as previously described^66^. In brief, cell suspensions from brain tissues were fractionated on 30/60% Percoll gradients at 1,000 *g* for 25 min. Astrocytes were collected from the PBS/30% interface. The purity of the isolated astrocytes was determined by FACS analysis using an anti-GFAP antibody (Cell Signaling Technology, #3670, 1:500).

### Glial cells and neuron-enriched mesencephalic cultures

Mouse primary mixed glial cells were cultured from the cerebral cortices of 1- to 3-day-old mice, as described in our previous study^67^. The proportion of microglia in murine mixed glial cultures was demonstrated to be 30–50% by FACS analyses using an anti-CD11b antibody (eBioscience, 11-0112, 5 μg ml^−1^). Neuron-enriched mesencephalic cells were cultured from embryonic day 14 mice as described previously^67^. In brief, ventral mesencephalic tissues were dissected and incubated in Ca^2+^-, Mg^2+^-free HBSS (CMF-HBSS) for 10 min and a 0.01% trypsin in CMF-HBSS for 9min at 37°C. The cultures were rinsed twice in Dulbecco's modified eagle's medium (DMEM) supplemented with 10% fetal bovine serum, 6mgml^−1^ glucose, 204μg ml^−1^ L-glutamine and 100U ml^−1^ penicillin/streptomycin (P/S) for trypsin inhibition, and then dissociated into single cells by trituration. Cells were seeded onto plates (2 × 10^6^ cells per well) precoated with poly-D-lysine (5 mg ml^−1^) and laminin (0.2 mg ml^−1^).

### Adenoviral transduction

A nonreplicative adenovirus (AD-GFP/Cre) in which the Cre recombinase gene is expressed under the control of the cytomegalovirus promoter was purchased from Vector Biolabs. A reporter Ad-GFP was used as the control (Vector Biolabs). For adenoviral transduction, primary mixed glial cells were cultured from *HIF1-α*^*+f/+f*^ mice, and infected with Ad-GFP or Ad-GFP/Cre (multiplicity of infection (MOI)=100) for 24 h. Infection efficiency, assessed by flow cytometry, was determined to be ~50%.

### ChIP assay

ChIP assay was performed using a ChIP assay kit (Upstate Biotechnology). Mouse primary mixed glial cells were incubated under hypoxic conditions for 24 h, and immediately fixed with 1% formaldehyde/phosphate-buffered saline, and sonicated to obtain 500- to 1,000-bp DNA fragments. Chromatin was immunoprecipitated with 5 μg of anti-HIF-1α (Novus, NB100-134) or rabbit IgG. The immunoprecipitated DNA was amplified with a promoter pair specific for the TIM-3-promoter (F, 5′-CCTGCTGCTTTGGAATTTGC-3′; and R, 5′-GAGTACTTGGCAGGGGAAATC-3′).

### Neutrophil migration assay

Neutrophils were isolated with a FACS Aria system (BD Bioscience), based on binding of FITC-conjugated anti-CD11b (eBioscience, 11-0112, 5 μg ml^−1^) and PE-conjugated anti-Gr-1(Ly6G) (eBioscience, 12-5931, 2 μg ml^−1^). Sorted neutrophils were added to the upper chamber of Transwell inserts positioned on 24-well plates in which mouse primary mixed glial cells had been seeded. The cells were incubated under 1% O_2_ or 20% O_2_ for 24 h, and transmigration was evaluated using a haematocytometer and flow cytometry.

### Determination of neurological deficits

Neurological deficit was assessed by a neurological scoring system according to a widely used method as follows^68^. The neurological scores of mice were given as follows: 0, normal motor function; 1, flexion of contralateral torso and forelimb upon lifting by tail; 2, circling to the contralateral side when mouse was held by the tail, but normal posture at rest; 3, leaning to contralateral side at rest and 4, no spontaneous motor activity.

### Immunohistochemistry

For immunohistochemistry, brains were removed, postfixed and embedded in paraffin. Coronal sections (10-μm thick) through the infarct were cut using a microtome and mounted on slides. The paraffin was removed, and the sections were washed with PBS-T and blocked in 10% bovine serum albumin for 2 h. Thereafter, the following primary antibodies were applied: goat anti-TIM-3 (Santa Cruz Biotechnology, sc-30326, 2 μg ml^−1^), rat anti-Gr-1(Ly6G) (eBioscience, 14-5931, 10 μg ml^−1^), rat anti-neutrophil (Abcam, ab2557, 2 μg ml^−1^), rabbit anti-MPO (Dako, A0398, 10 μg ml^−1^), rabbit anti-Iba-1 (Wako, #019-19741, 2 μg ml^−1^), rabbit anti-cleaved caspase-3 (Cell Signaling Technology, #9662S, 1:300), mouse anti-NeuN (Millipore, #MAB377, 10 μg ml^−1^). Hypoxic regions were detected using pimonidazole (Hypoxyprobe-1, Natural Pharmacia International) as described previously^69^. Images were obtained using a confocal microscope (Carl Zeiss LSM510). For assessment of TIM-3 expression in primary glial cells, mouse primary mixed glial cells were fixed with methanol, washed with PBS-T and incubated at 4 °C with anti-TIM-3 antibody (R&D Systems, AF1529, 1 μg ml^−1^).

### TIM-3 promoter assay

A 1,517-bp fragment of the mouse TIM-3 promoter (−1,517 to +1 relative to the start codon) was PCR-amplified from genomic DNA and cloned into the PGL3 basic vector (Promega). Site-directed mutagenesis of each HRE was performed using mutant primers and Phusion High-Fidelity DNA Polymerase (NEB). All constructs were confirmed by DNA sequencing. Mouse primary mixed glial cells were transfected using Lipofectamine 2000 (Invitrogen) according to the manufacturer’s instructions. After transfection, cells were incubated under 1% O_2_ or 20% O_2_ for 24 h, and reporter gene activity was determined with a luciferase assay system (Promega). β-Galactosidase activity was measured for the normalization of transfection efficiency.

### Western blot analysis

Right and left hemispheres were dissected from H/I mice, and homogenized with a pellet pestle (Fisher) in ice-cold RIPA buffer containing protease inhibitors (2 mM phenylmethylsulphonyl fluoride, 100 μg ml^−1^ leupeptin, 10 μg ml^−1^ pepstatin, 1 μg ml^−1^ aprotinin and 2 mM EDTA). Homogenates were centrifuged at 12,000 r.p.m. for 30 min at 4 °C, and supernatants were collected. The samples were separated by SDS–polyacrylamide gel electrophoresis, transferred to nitrocellulose membranes and incubated with the following primary antibodies: goat anti-TIM-3 (R&D Systems, AF1529, 0.1 μg ml^−1^), mouse anti-PARP (Zymed, 33-3100, 2 μg ml^−1^), rabbit anti-MPO (Dako, A0398, 2 μg ml^−1^), goat anti-Iba-1 (Abcam, ab5076, 0.5 μg ml^−1^), mouse anti-GFAP (Cell Signaling Technology, #3670, 1:1,000), mouse anti-NeuN (Millipore, #MAB377, 1 μg ml^−1^), mouse anti-α-tubulin (Sigma, T5168, 1:5,000), microtubule-associated protein 2 (Millipore, #MAB3418, 1 μg ml^−1^), glutamate decarboxylase (Abcam, ab11070, 1 μg ml^−1^), peroxidase-conjugated goat anti-rabbit (Bio-Rad, #170-6515, 1:5,000), peroxidase-conjugated rabbit anti-goat (Zymed, R-21459, 1:5,000), peroxidase-conjugated goat anti-mouse (Bio-Rad, #170-6516, 1:5,000). The results were visualized using an enhanced chemiluminescence system, and quantified by densitometric analysis (Image J software, NIH). All experiments were performed independently at least three times.

### RT–PCR analysis

Total RNA was isolated using Easy-Blue (iNtRON), and cDNA was synthesized using avian myeloblastosis virus reverse transcriptase (TaKaRa) according to the manufacturer’s instructions. PCR was performed with 25–30 cycles of sequential reactions. All experiments were performed independently at least three times, and the PCR products were quantified using NIH Image J and normalized to actin. The QuantiFast SYBR Green PCR kit (Qiagen) was used for real-time PCR. Roche LightCycler 480 Real-Time PCR System (Roche Applied Science) and LigthCycler 480 Quantification Software Version 1.5 were used for real-time PCR performance and analysis. The primers used in quantitative PCR were as follows: (forward) 5′-GGATGAGGACATGAGCACCT-3′ and (reverse) 5′-TCCATTGAGGTGGAGAGCTT-3′ for IL-1β; (forward) 5′-TGCACCCAAACCGAAGTCAT-3′ and (reverse) 5′-TTGTCAGAAGCCAGCGTTCAC-3′ for CXCL1; (forward) 5′-CTCATCAGTTGCCACTTCC-3′ and (reverse) 5′-TCATCTTCACTGTCTAGACCAC-3′ for HIF-1α; (forward) 5′-TGTCGTGGAGTCTACTGGTGTCTTC-3′ and (reverse) 5′-CGTGGTTCACACCCATCACAA-3′ for GAPDH. The sequences of the utilized PCR primers were follows: (forward) 5′-CCCTGCAGTTACACTCTACC-3′ and (reverse) 5′-GTATCCTGCAGCAGTAGGTC-3′ for TIM-3; (forward) 5′-AGCCTTAACCTGTCTGCCACTT-3′ and (reverse) 5′-GAAATCATTTAACATTGCATATATACTAGAACAT-3′ for HIF1α; (forward) 5′-AGGATAGGACTGGATTTGCCTG-3′ and (reverse) 5′-GTGGTGATGCCAGTGTTGTCA-3′ for MPO; (forward) 5′-TACAGGCTCCGAGATGAACAACAA-3′ and (reverse) 5′-TGGGGAAGGCATTAGAAACAGTCC-3′ for IL-1β; (forward) 5′-CGCTCGCTTCTCTGTGCAGC-3′ and (reverse) 5′-GTGGCTATGACTTCGGTTTGG-3′ for CXCL1; (forward) 5′-CATGTTTGAGACCTTCAACACCCC-3′ and (reverse) 5′-GCCATCTCCTGCTCGAAGTCTAG-3′ for Actin.

### Flow cytometry

All staining steps were performed in the dark and blocked with BD Fc Block. Freshly obtained microglia and astrocytes were stained using the following antibodies: rabbit anti-Iba-1 (Wako, #019-19741, 1 μg ml^−1^) followed by Alexa 488-conjugated chick anti-rabbit (Invitrogen, A21441, 2 μg ml^−1^), and either PE-conjugated anti-mouse TIM-3 (eBioscience, RMT-3-23, 2 μg ml^−1^) or isotype control Ab (eBioscience, 2 μg ml^−1^) for 30 min at 4 °C. For intracellular staining of GFAP, cells were fixed and permeabilized for 20 min with IC fixation/permeabilization buffer (eBioscience), washed twice with permeabilization buffer, incubated with anti-GFAP (Cell Signaling Technology, #3672, 1:500) in permeabilization buffer for 30 min and stained with Alexa 488-conjugated chick anti-mouse (Invitrogen, A21200, 2 μg ml^−1^). The data were analysed with the CellQuest software (BD Bioscience) and FlowJo software (Treestar) packages.

### Lentiviral production and stereotaxic injection

The coding sequence of TIM-3 (GE Dharmacon) was ligated into the PLL3.7.EF1α plasmid (Addgene, Inc.) to produce PLL3.7.EF1α-TIM3. The plasmid was then used to generate the recombinant lentivirus LV-TIM3-GFP. As a control, a lentiviral vector that expressed GFP alone (LV-GFP) was generated. Lentiviruses were titrated using flow cytometry as previously reported^70^. LV-TIM3-GFP or LV-GFP was injected using a stereotaxic instrument. Each mouse received four intracranial injections of lentivirus (20 μl containing 5 × 10^6^ TU ml^−1^ into the right hemisphere). For *in vitro* fluorescence imaging, the collected cells were analysed by FACS and western blotting using an anti-GFP antibody (Santacruz, sc-9996, 1:1,000). Whole-body *in vivo* imaging was performed in a fluorescent light box illuminated at excitation filter, from 445 to 490 nm, and emission filter, from 515 to 575 nm, using Caliper Life Science’s Xenogen IVIS Spectrum.

### Data analysis

All data are expressed as the mean±s.d. *Post-hoc* comparisons (Student–Newman–Keuls test) were performed using SigmaPlot 10.0. Neurological scores were assessed by nonparametric statistical procedures. Two group (IgG versus anti-TIM-3, *HIF-1α*^*+f/+f*^ mice versus *LysM-HIf-1α*^−*/*−^ mice, LV-GFP injected *LysM-HIf-1α*^−*/*−^ mice versus LV-TIM3-GFP injected *LysM-HIf-1α*^−*/*−^ mice) comparisons were analysed by the Mann–Whitney *U*-tests.

## Author contributions

E.J.P. designed the research; H.S.K., C.Y.C., H.J.Y., Y.-H.A., H.-S.K. and S.-B.J. performed the experiments; S.H.J., R.S.J., H.S.K., I.-H.K. and E.J.P. analysed the data; H.S.K. and E.J.P. wrote the paper.

## Additional information

**How to cite this article:** Koh, H. S. *et al.* The HIF-1/glial TIM-3 axis controls inflammation-associated brain damage under hypoxia. *Nat. Commun.* 6:6340 doi: 10.1038/ncomms7340 (2015).

## Supplementary Material

Supplementary Information Supplementary Figures 1-11

Supplementary Movie 1 Leaning behavior of HIF-1a+f/+f mouse 24 h after H/I.

Supplementary Movie 2 Circling behavior of HIF-1a+f/+f mouse 24 h after H/I on a flat surface.

Supplementary Movie 3 Motor behavior of LysM-Hif-1a-/- mouse 24 h after H/I on a flat surface.

Supplementary Movie 4 Motor behavior of LysM-Hif-1a-/- mouse 24 h after H/I on a flat surface.

## Figures and Tables

**Table 1:** Neurological deficit scores in mice after 24 h of H/I in mice with IgG (*n*=5) or anti-TIM-3 (*n*=6).

**Treatment**	**Number with the indicated score**					***n***	**Mean±s.d.**
	**0**	**1**	**2**	**3**	**4**		
IgG control	0	0	2	2	1	5	2.8±0.8
TIM-3-blocking antibody	3	2	1	0	0	6	0.8±0.8*

H/I, hypoxia-ischaemia; TIM, T-cell immunoglobulin and mucin domain protein.

The data were analysed by Mann–Whitney *U-*test; ^*^P=0.012.

**Table 2:** Neurological deficit scores after H/I in HIF-1α^+f/+f^ (*n*=10) and LysM-Hif-1α^−/−^ (*n*=11) mice Mean scores are given±s.d.

**Mouse**	**Number with the indicated score**					***n***	**Mean±s.d.**
	**0**	**1**	**2**	**3**	**4**		
*HIF-1α*^*+f/+f*^	0	2	3	2	3	10	2.6±1.1
*LysM-HIf-1α*^−*/*−^	6	4	1	0	0	11	1.2±0.6**

H/I, hypoxia-ischaemia.

The data were analysed by Mann–Whitney *U-*test; **P=0.0008.
