# Supplementary material for: The HIF-1/glial TIM-3 axis controls inflammation-associated brain damage under hypoxia
Source: Nat Commun. 2015 Mar 20;6:6340. doi: 10.1038/ncomms7340 (PMC4383004; doi:10.1038/ncomms7340)
Supplement: Supplementary Information — Supplementary Figures 1-11 [file ncomms7340-s1.pdf]

Supplementary Figure 1

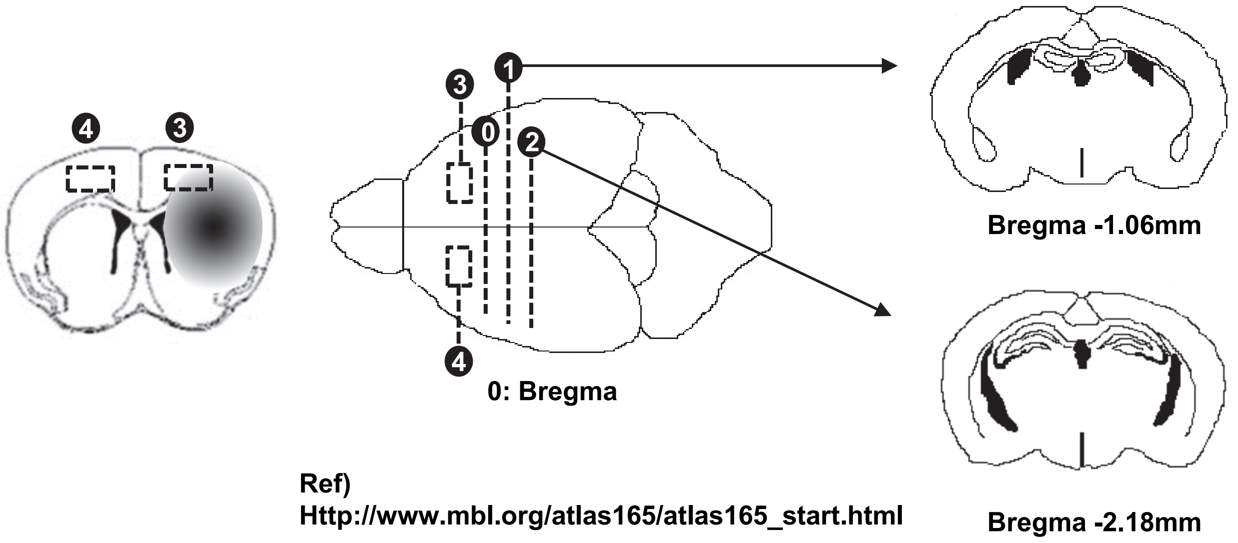

Supplementary Figure 1| Schematic representation of the utilized brain slice for TTC staining and immunohistochemistry, which was located at -1.06 mm and -2.18 mm in adult mice (Modified from the atlas of Franklin and Paxinos[38]. Confirming H/I model by TTC staining, the brain tissues from the boxed ipsilateral penumbra (③) and contralateral area (④) were used for RT-PCR and Western blot analyses.

Supplementary Figure 2

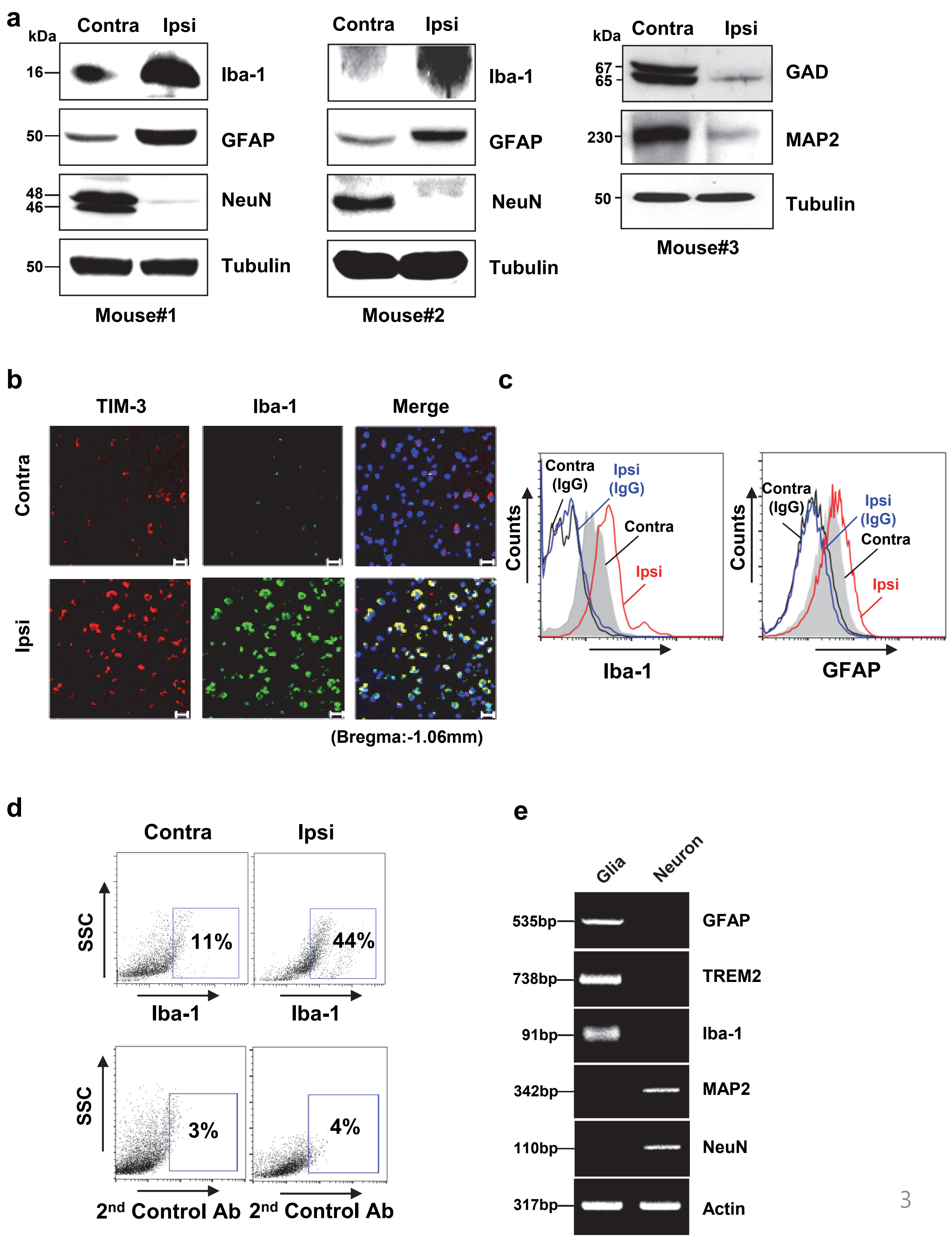

Supplementary Figure 2│ Expression levels of glial or neuronal markers in brain tissues from the H/I mice and primary culture cells. (a) Western blot analysis was performed on brain tissues from the H/I mice using the indicated antibodies. **(b)** Immunohistochemistry was performed with Anti-TIM-3 (red) and Iba-1 antibodies (green) on coronal sections of the H/I mice. Scale bar: 20 μm. **(c-d)** Brain cells isolated from ischemic ipsilateral and contralateral hemispheres were stained with antibodies against Iba-1, GFAP, or secondary antibody (Molecular probe, #A21441), and analyzed by flow cytometry. The data shown are representative of at least three independent experiments. **(e)** RT-PCR analysis was performed in primary cultured glial cells and neuronal cells using the indicated primers. GFAP forward: 5`-AAG CTC CAA GAT GAA ACC AAC CTG A-3`, reverse: 5`-CCA CGA TGT TCC TCT TGA GGT GG-3`; and TREM2 forward: 5`-ATG GGA CCT CTC CAC CAG TT, reverse: 5`-TCA CGT ACC TCC GGG TCC A; and Iba-1 forward: 5`-GGA TTT GCA GGG AGG AAA AG-3`, reverse: 5`-TGG GAT CAT CGA GGA ATT G-3`; and MAP2 forward: 5`-TTG GCT CAC TTG ACA ATG CTC ACC-3`, reverse: 5`-AAT ATG ACA CCT GCT CAG AGC CCA-3`; and NeuN forward: 5`-CCA GGC ACT GAG GCC AGC ACACAG C-3`, reverse: 5`-CTC CGT GGG GTC GGA AGG GTG G-3`.

Supplementary Figure 3

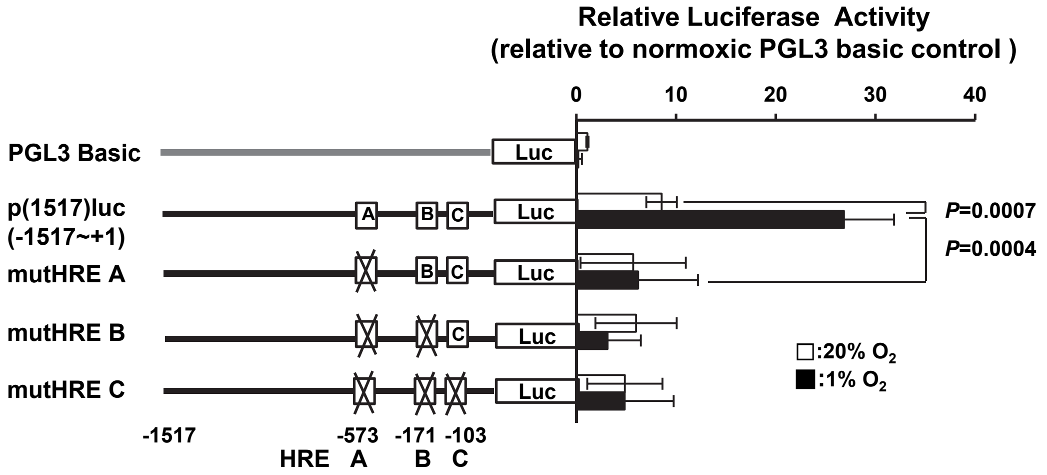

Supplementary Figure 3│ TIM-3 expression is closely linked with HIF-1α Three putative HRE sites within p(1517)luc were targeted for mutagenesis. The mutations of 2 base pairs in each of the HREs were made using a site-directed mutagenesis kit, and confirmed using DNA sequencing. The regions of mutated HREs are indicated at the bottom. Mouse primary mixed glial cells were transfected with the TIM-3 luciferase reporter constructs or PGL3-basic control reporter, and incubated under hypoxic or normoxic conditions for 24 h. Relative promoter activity is expressed as the ratio of luciferase activity/β-galactosidase activity.

Supplementary Figure 4

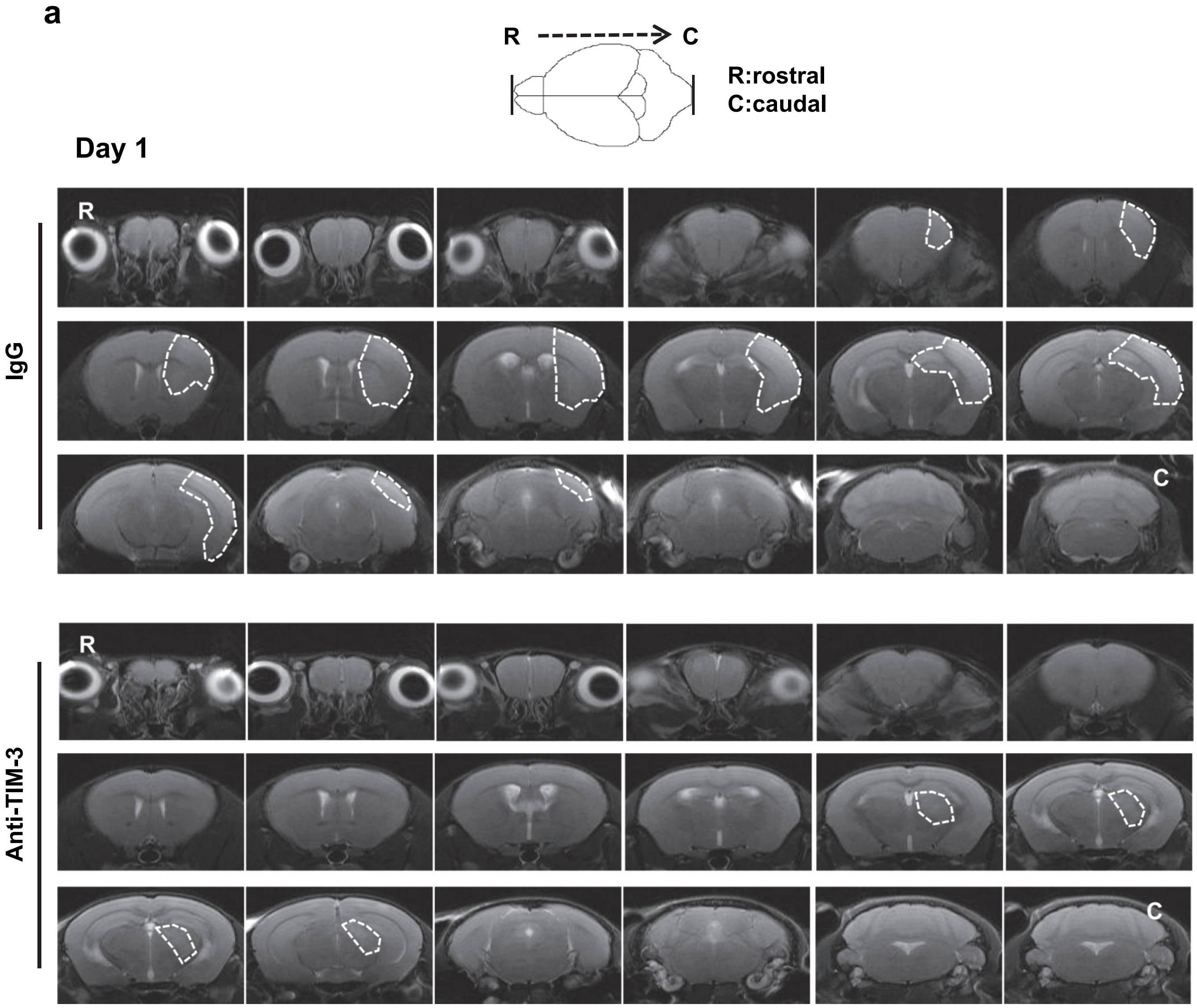

Supplementary Figure 4 (continued)

b

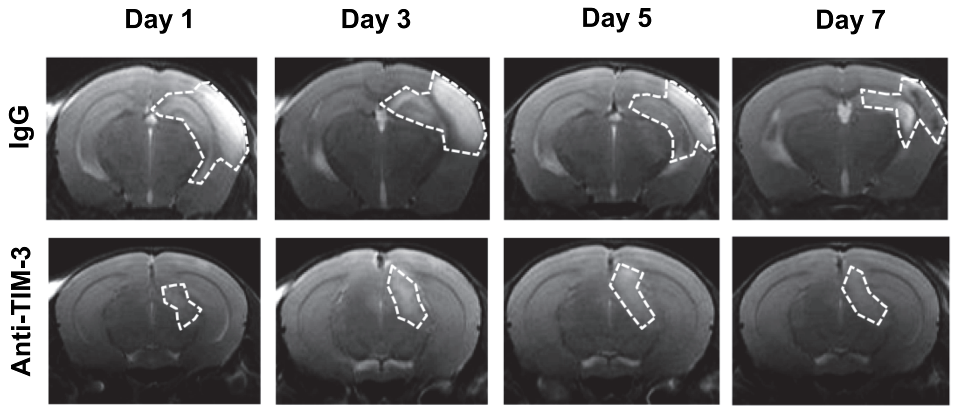

c

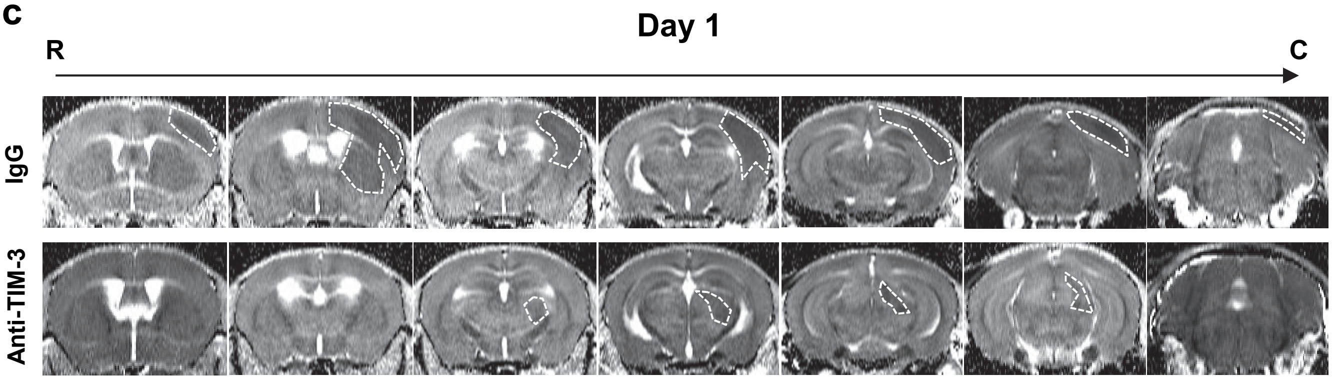

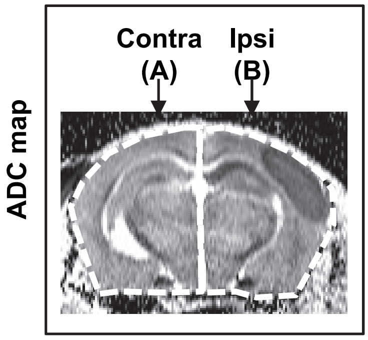

% of edema:

$$\frac{\text{Ipsilateral volume (B)} - \text{Contralateral volume (A)}}{\text{Contralateral volume (A)}} \times 100$$

Supplementary Figure 4| A TIM-3 blocking antibody significantly reduces brain injury and edema formation after hypoxia-ischemia (a) Representative images of Coronal MRI (T2 weighted image) brain slices after 24 h from H/I mice treated with 100 µg of IgG (*n*=4) or anti-TIM-3 (*n*=4). **(b)** Representative images of Coronal MRI (T2 weighted image) brain slices after indicated time from H/I mice treated with 100 µg of IgG (*n*=4) or anti-TIM-3 (*n*=4). **(c)** Representative images of ADC maps brain slices after 24 h from H/I mice treated with 100 µg of IgG (*n*=4) or anti-TIM-3 (*n*=4). Edema volume was quantified with Image J analyzer and were calculated with [(ipsilateral volume-contralateral volume)/contralateral volume] × 100 and expressed as a percentage of the damaged ipsilateral hemisphere.

Supplementary Figure 5

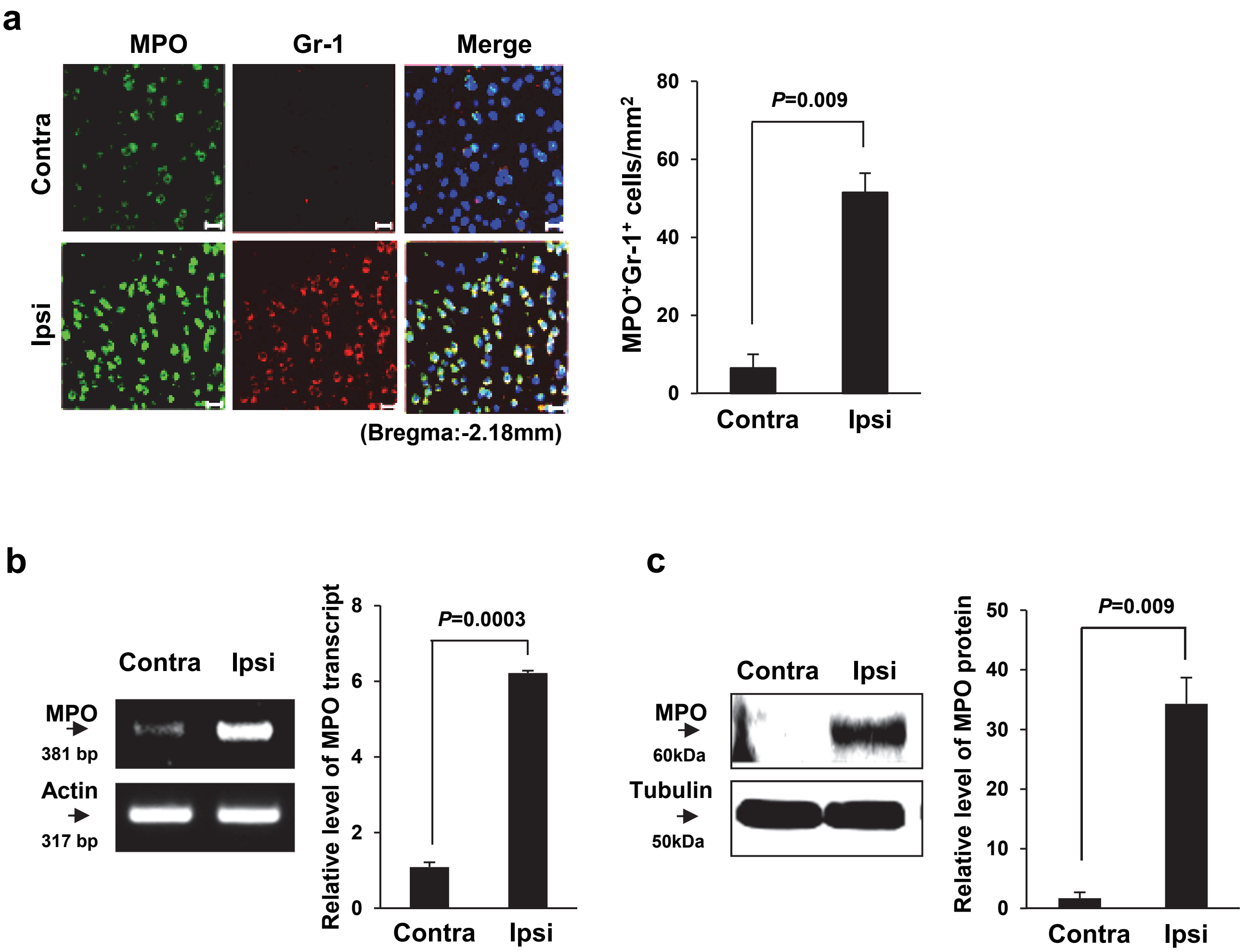

Supplementary Figure 5| Neutrophil migration is increased in the ipsilateral cortex of H/I mice. **(a)** Antibodies against MPO and Gr-1 were used to perform immunohistochemistry on coronal sections of the H/I mice 24 h after injury. Scale bar: 20 µm. The graph shows the mean number of $MPO^{+}Gr\text{-}1^{+}$ cells per mm^2 (mean ± s.d. from three independent experiments). **(b)** RT-PCR analysis and **(c)** Western blot analysis were performed on brain tissues from H/I mice with MPO-specific primers and antibody, respectively. Relative levels of MPO transcripts and proteins are shown as graphs. The data shown are representative of at least three independent experiments (**b**, p=0.0003 compared with the contralateral control; **c**, p=0.009 compared with the contralateral control).

Supplementary Figure 6

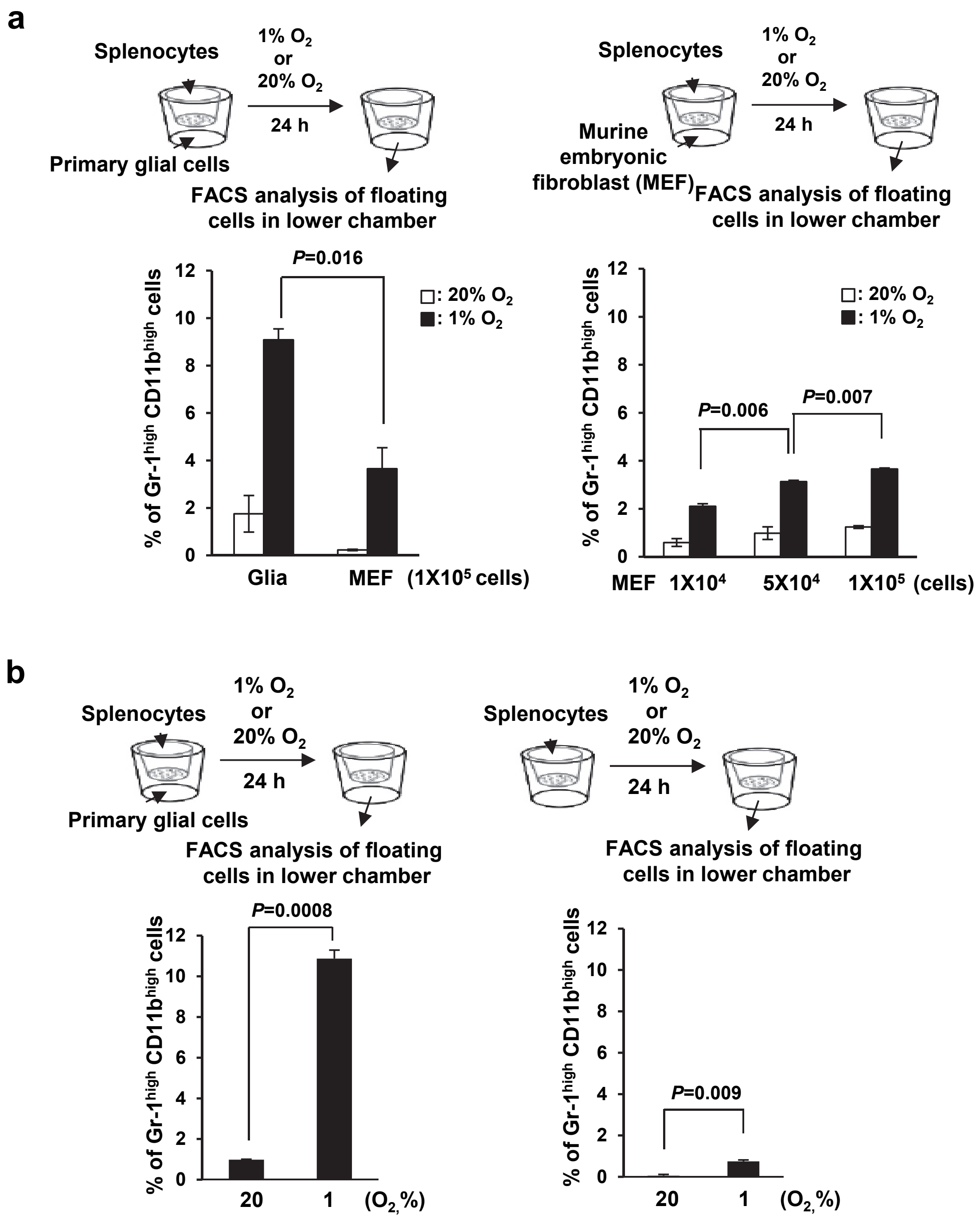

Supplementary Figure 6 (continued)

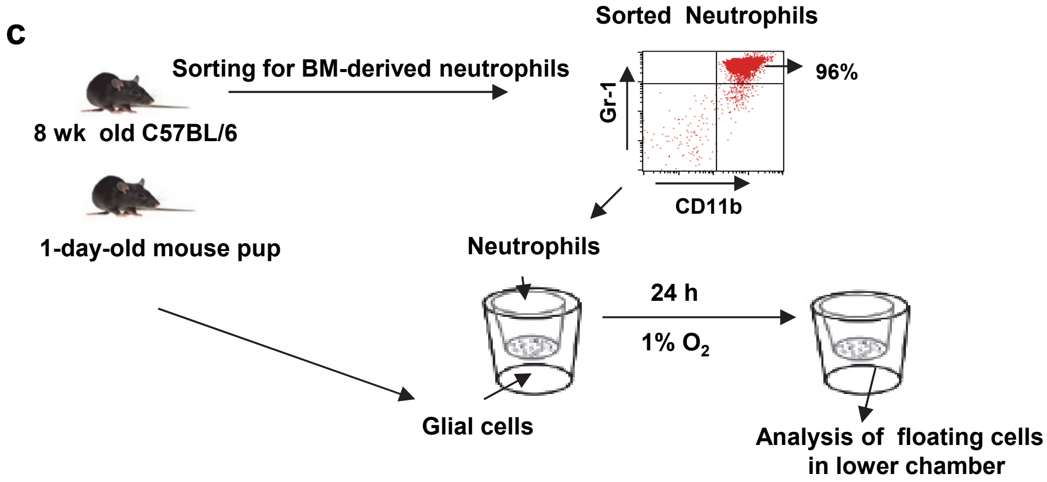

Supplementary Figure 6│ The migration of neutrophils is increased in the presence of glial cells under hypoxic conditions. (a) Splenocytes were isolated from C57BL/6 mice, and incubated in a transwell system with primary glial cells or MEF cells (C57BL/6) under hypoxic or normoxic conditions for 24 h. The cells were analyzed by flow cytometry using antibodies against CD11b and Gr-1. The percentage of Gr-1^{high}CD11b^{high} cells is expressed as the mean ± s.d. from three independent experiments. **(b)** Splenocytes (5×10^5) were isolated from C57BL/6 mice, and incubated in a transwell system with or without 2×10^5 primary cultured glial cells under hypoxic or normoxic conditions for 24 h. The percentage of Gr-1^{high}CD11b^{high} cells is expressed as the mean ± s.d. from three independent experiments. **(c)** Schematic illustration of our transwell system-based neutrophil transmigration assay.

Supplementary Figure 7

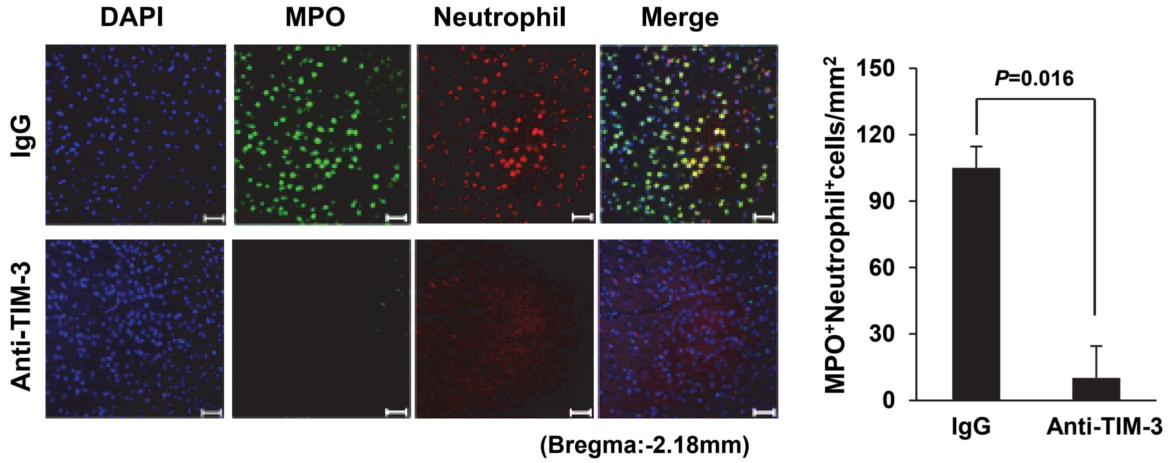

Supplementary Figure 7| The migration of neutrophils into ischemic ipsilateral regions is decreased in TIM-3-blocking antibody-treated H/I mice. Representative confocal microscopic images of coronal sections from control IgG- or TIM-3 blocking antibody-treated H/I mice. Scale bar: 50 µm.

Supplementary Figure 8

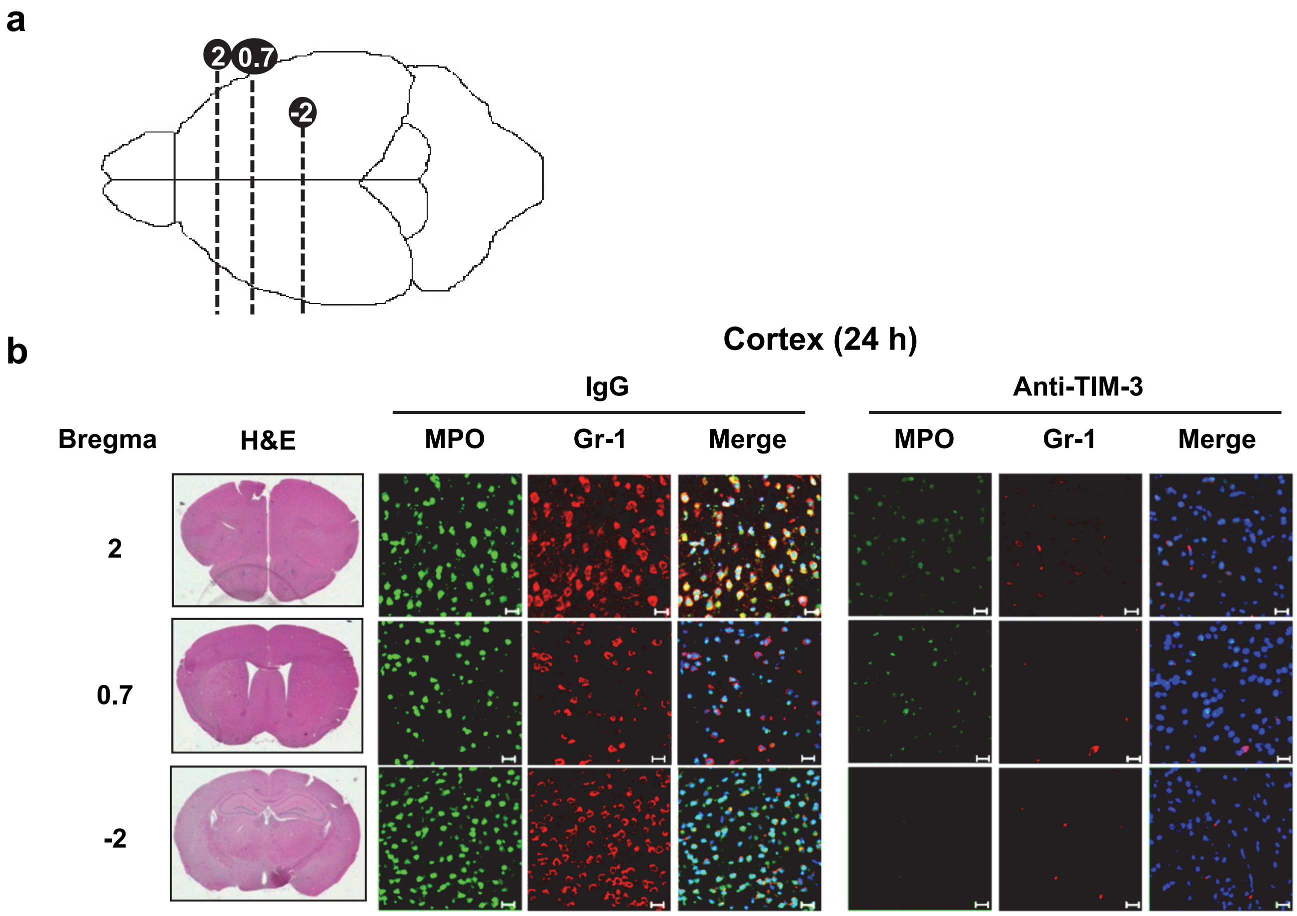

Supplementary Figure 8 (continued)

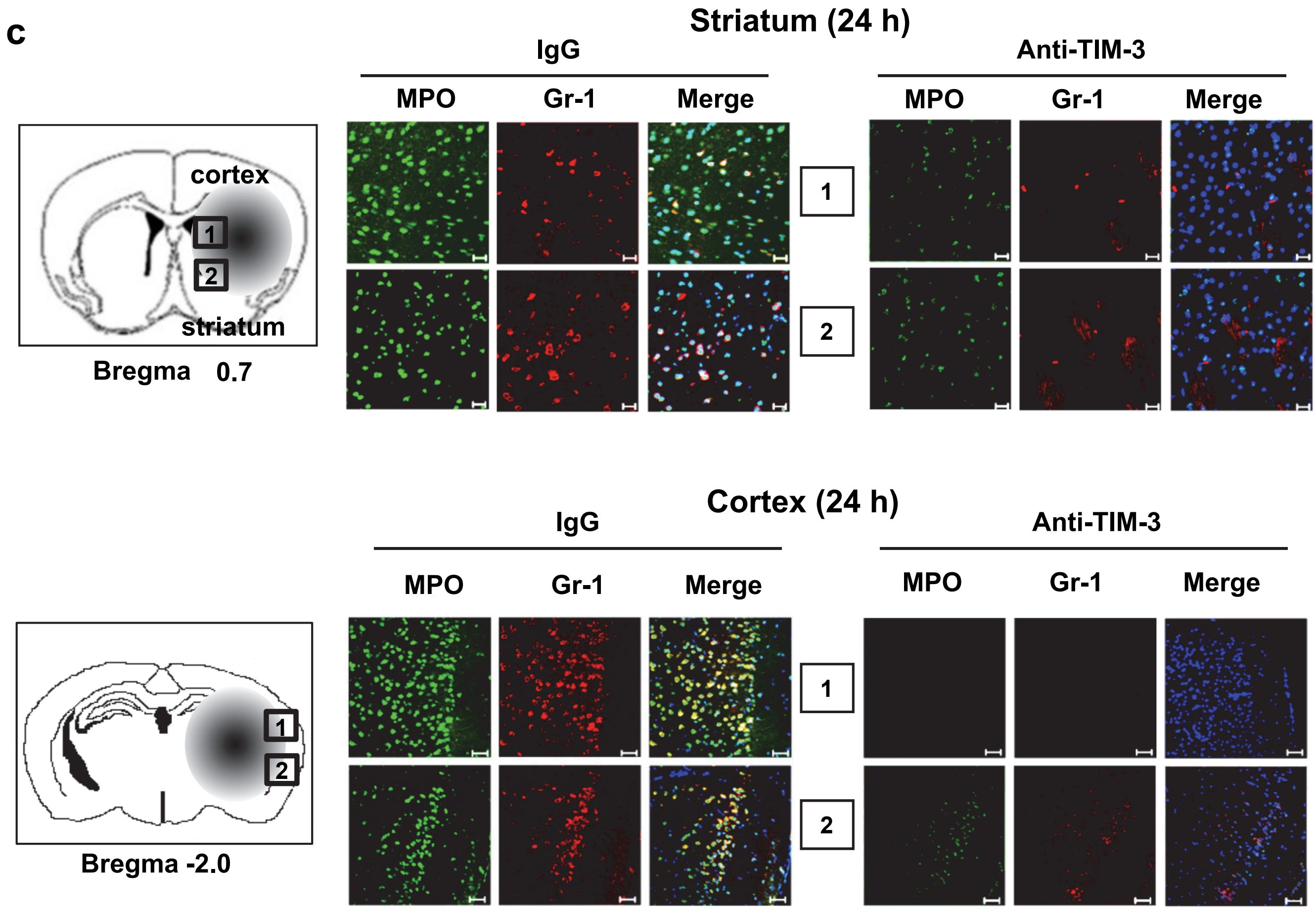

Supplementary Figure 8 (continued)

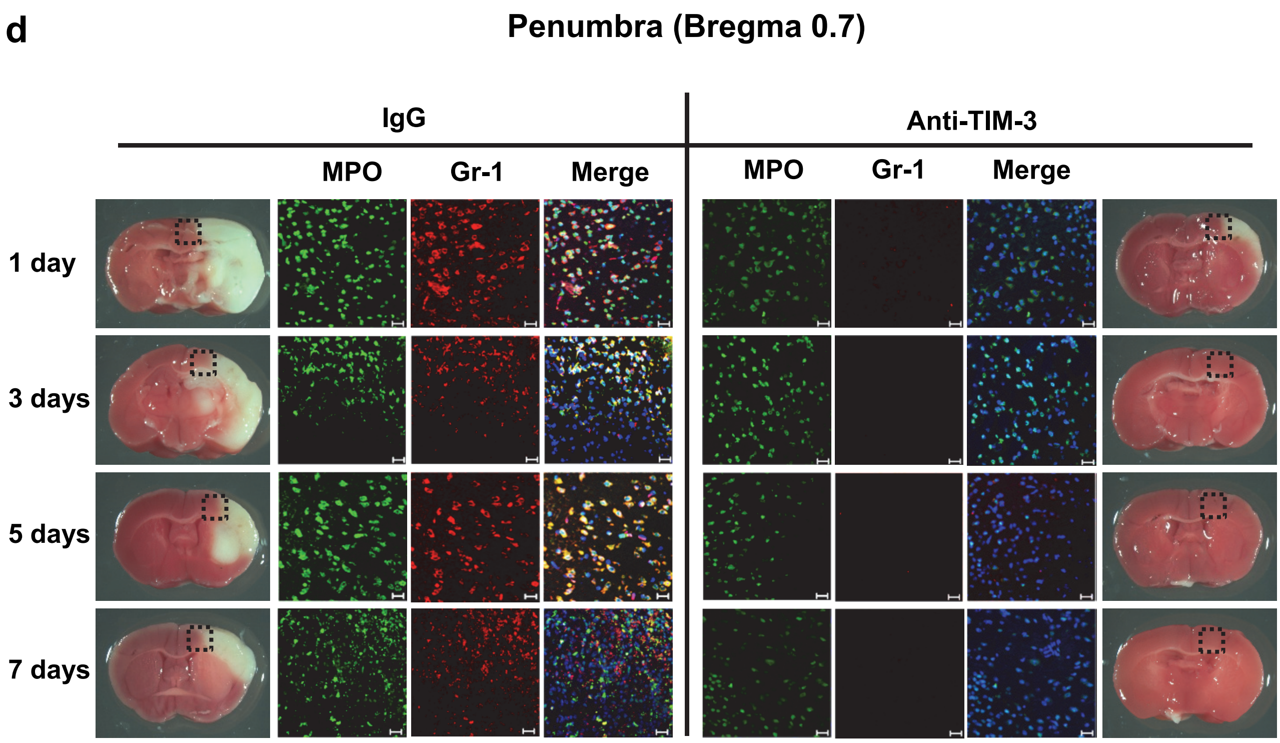

Supplementary Figure 8│ The TIM-3-blocking antibody reduces neutrophil migration after hypoxia-ischemia. **(a)** Schematic representation of the utilized brain slice for immunohistochemistry, which was located at bregma 2 to -2 mm in adult mice (Modified from the atlas of Franklin and Paxinos [38]). **(b-d)** Immunohistochemistry was performed on coronal sections from control IgG- or TIM-3-blocking-antibody-treated H/I mice at the indicated times after injury using antibodies against MPO and Gr-1. Scale bar: 20 μm.

Supplementary Figure 9

a

b

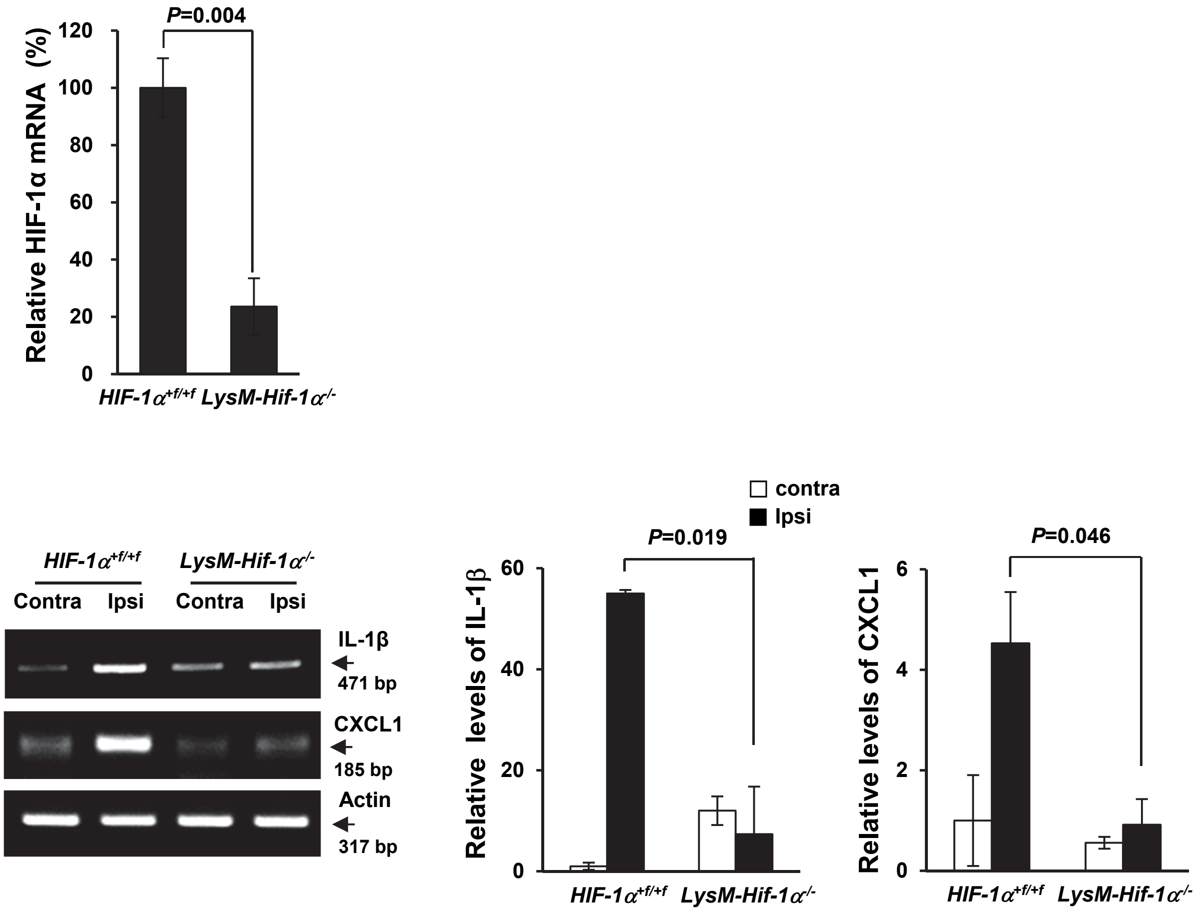

Supplementary Figure 9| Similar events are observed in the primary glial cells and cortex tissues from *LysM-Hif-1α$^{-/-}$* mice. **(a)** Real time quantitative RT-PCR analysis was performed in primary microglia cultured from *HIF-1α$^{+f/+f}$* or *LysM-Hif-1α$^{-/-}$* mice (*n*=6) using HIF-1α primer. **(b)** The transcript levels of IL-1β and CXCL1 were examined in brain tissues from the contralateral cortex and ischemic ipsilateral cortex of *HIF-1α$^{+f/+f}$* or *LysM-Hif-1α$^{-/-}$* mouse 24 h after hypoxia and ischemic injury (*n*=3). Data shown are presented as the mean ± s.d.

Supplementary Figure 10

a

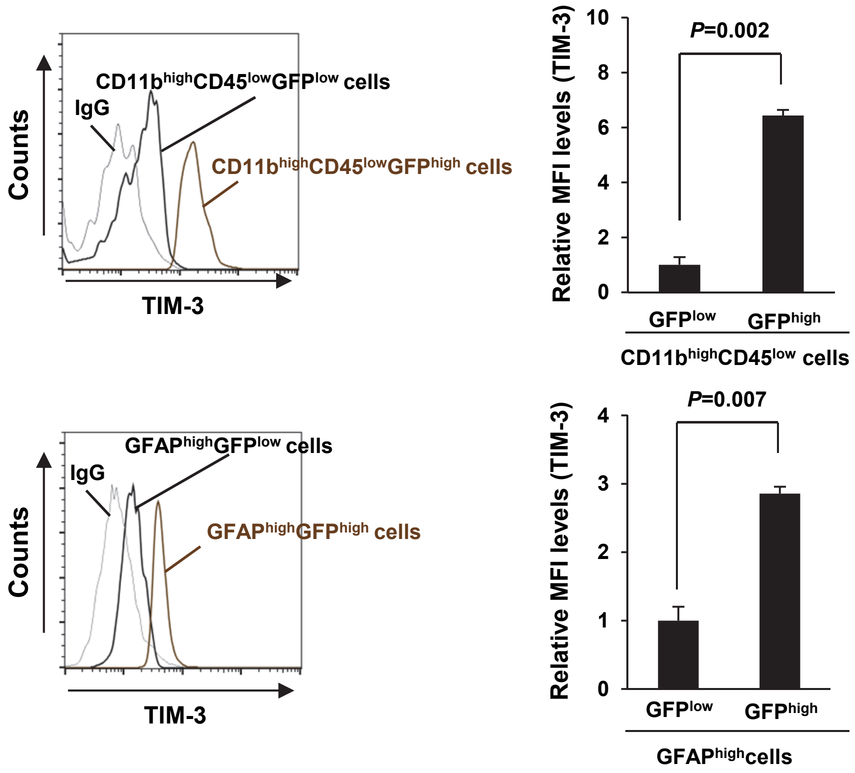

b

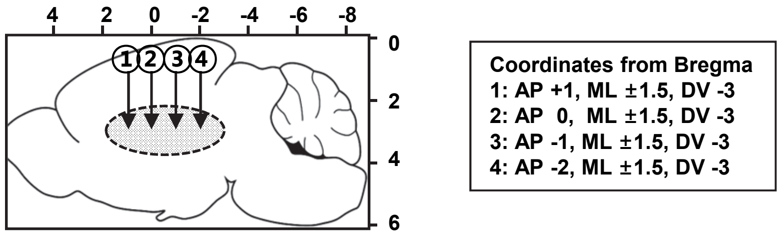

c

Mice	Lentivirus	Number with the indicated score					*n*	Mean Score (± s.d.)
		0	1	2	3	4		
LysM-Hlf-1α$^{-/-}$	**LV-GFP**	1	3	2	0	0	6	**1.1±0.7**
	**LV-TIM3-GFP**	0	1	2	3	0	6	**2.3±0.8***

Supplementary Figure 10| (a) LV-TIM3-GFP or LV-GFP was injected using an stereotaxic instrument. Five days after intracranial injection of lentiviral vectors, glial cells from the injected region were subjected to FACS analysis using antibody against TIM-3 (eBioscience, RMT-3-23). The graph shows the results from three mice. **(b)** Schematic drawing of the injection sites. **(c)** Neurological scoring was determined after 24 h of H/I in mice LV-GFP (*n*=6) or LV-TIM3-GFP (*n*=6).

Supplementary Figure 11

a Blots and Gels; Figure 1

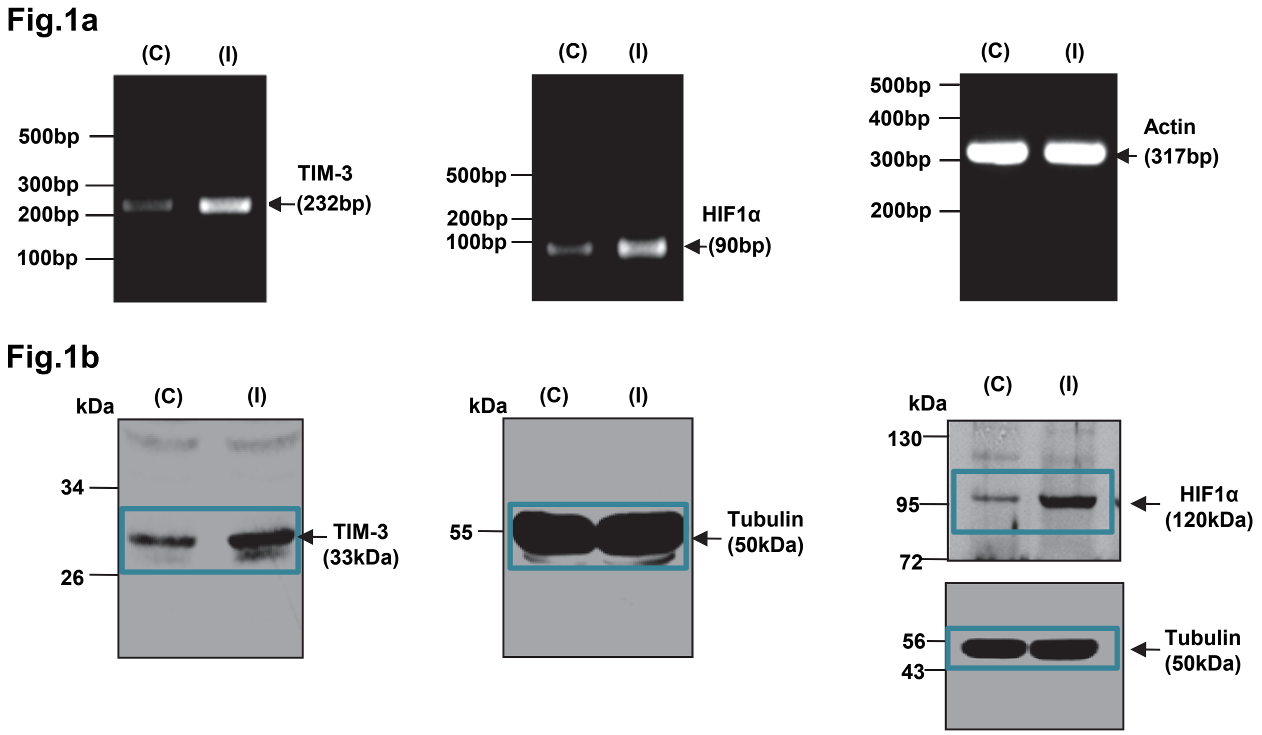

b Blots and Gels; Figure 2

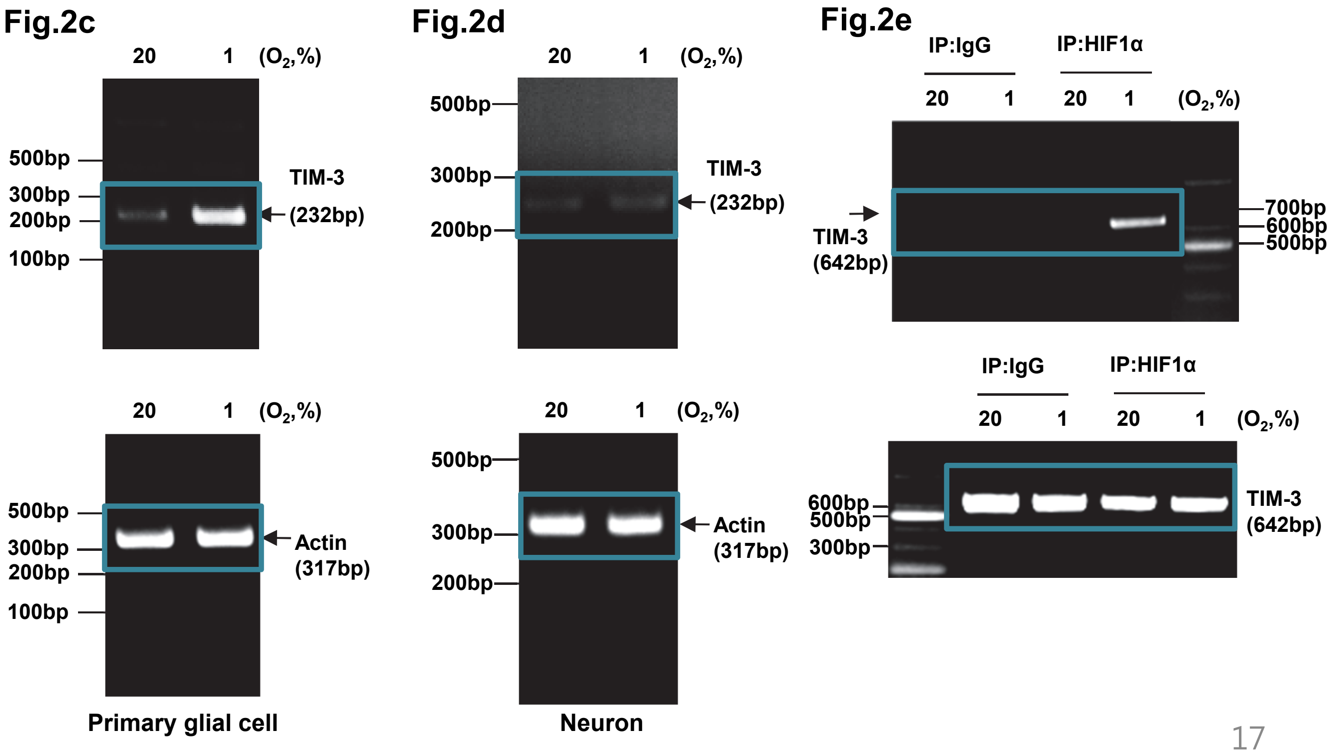

Supplementary Figure 11 (continued)

Fig.2g

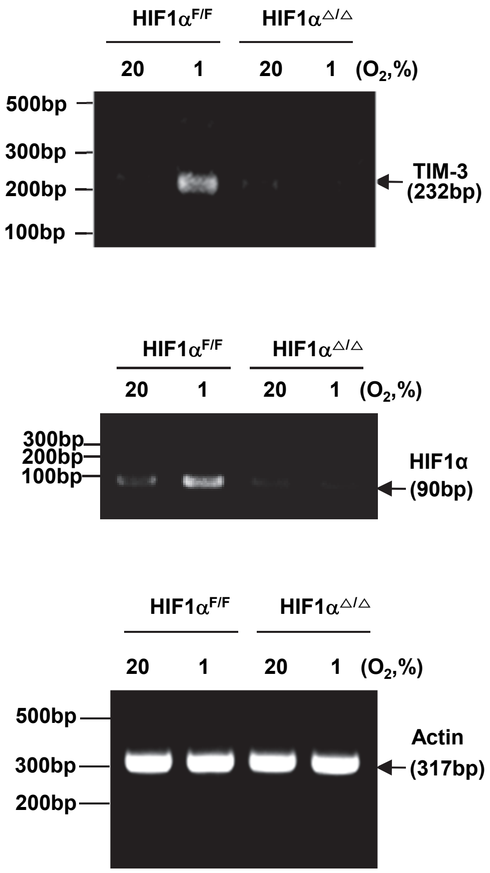

Fig.2h

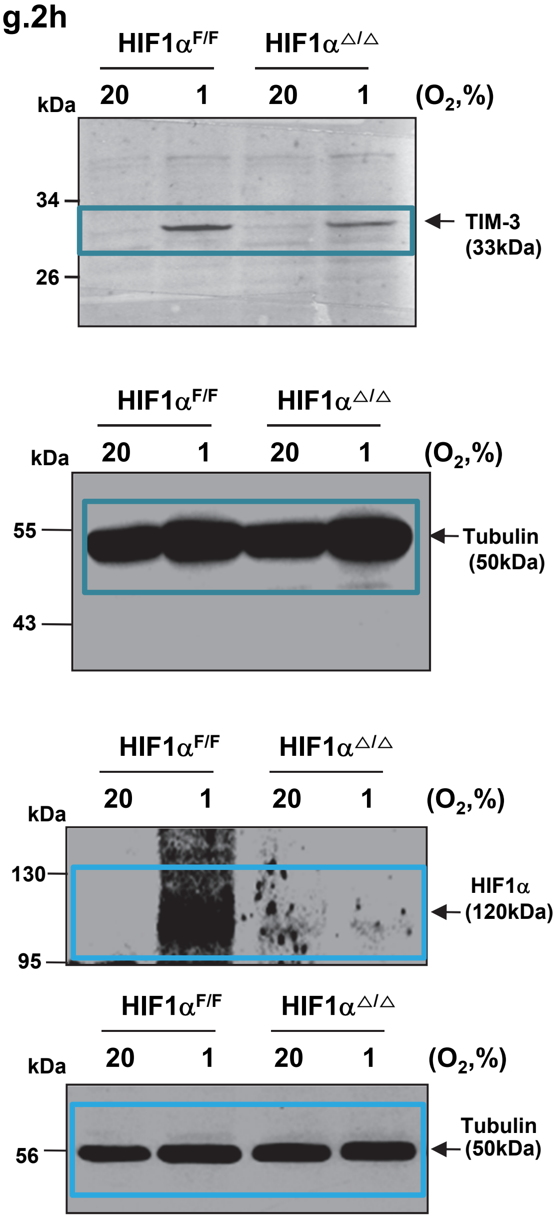

c <u>Blots and Gels; Figure 3f</u>

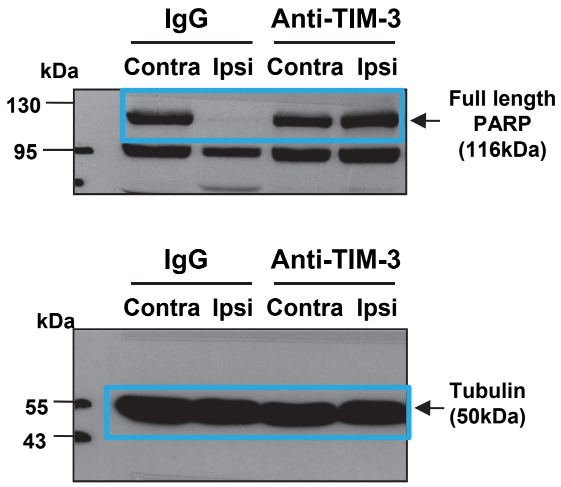

Supplementary Figure 11 (continued)

d Blots and Gels; Figure 4

Fig.4a

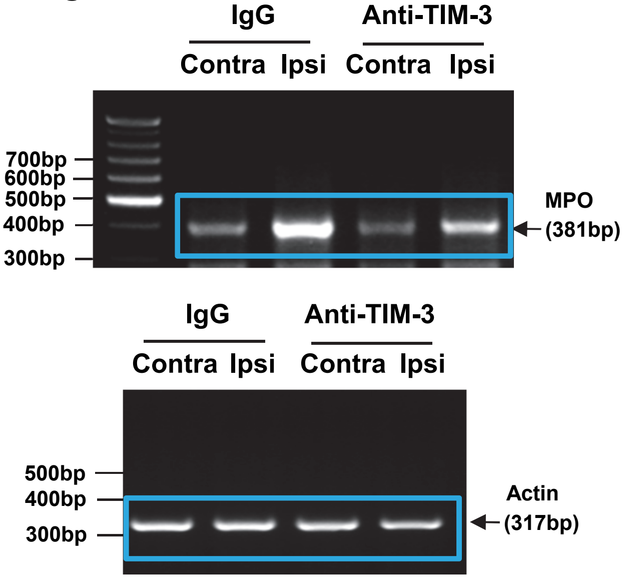

Fig.4b

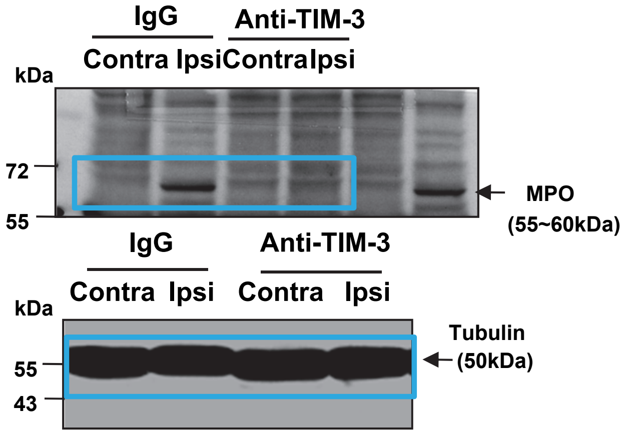

e Blots and Gels; Figure 5

Fig.5c

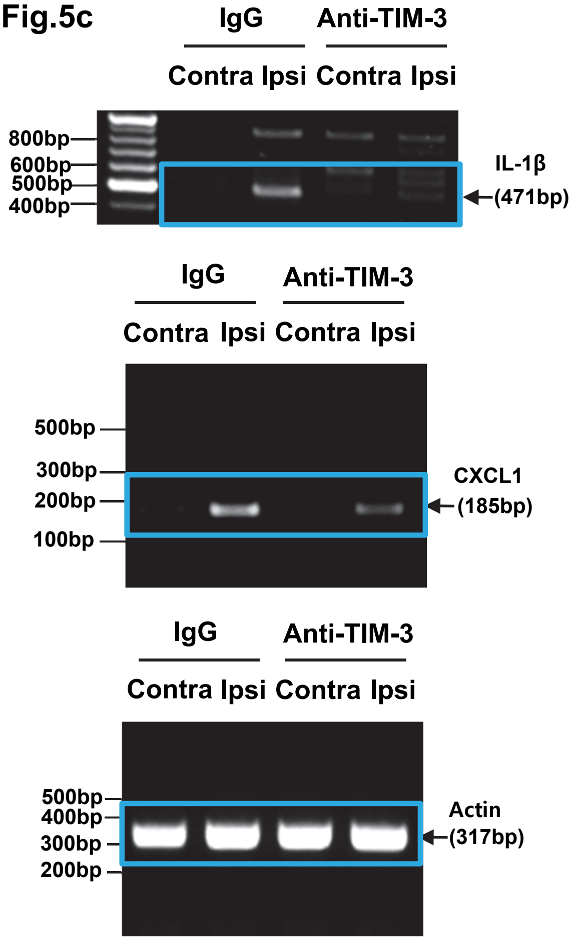

Fig.5e

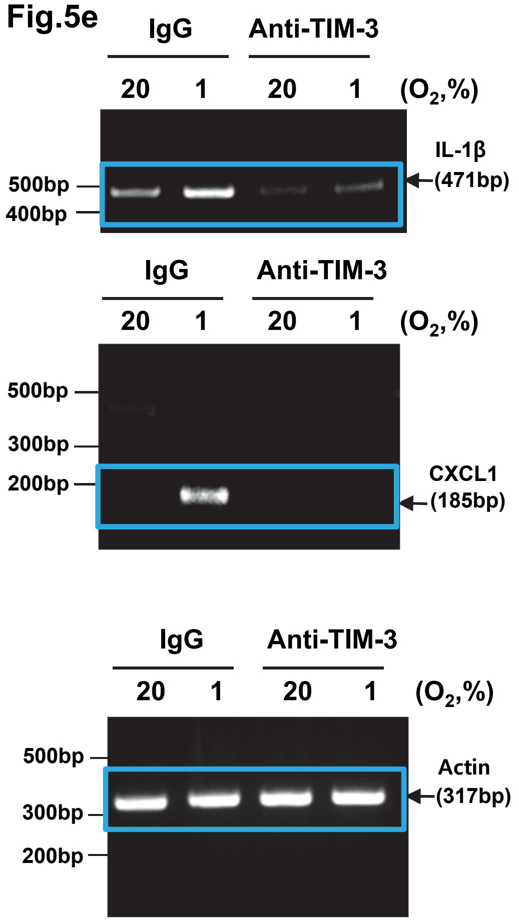

Supplementary Figure 11 (continued)

f Blots and Gels; Figure 6c

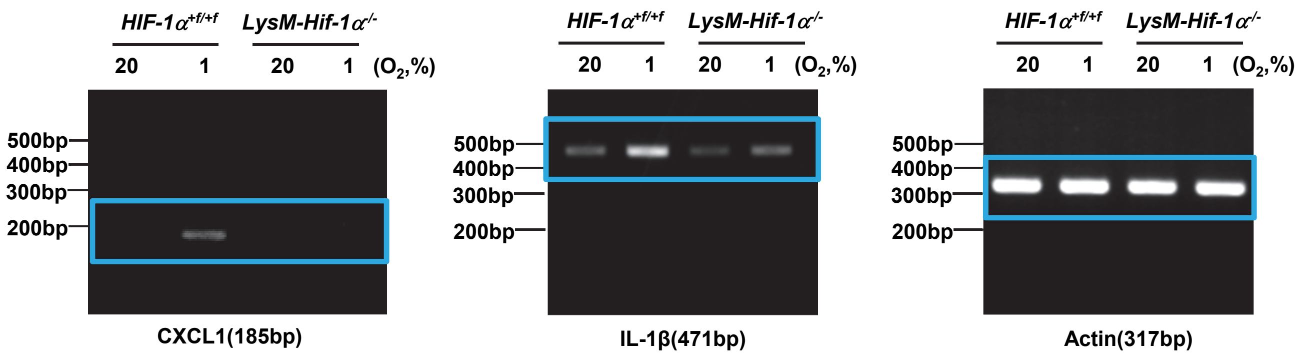

g Blots and Gels; Figure 7

Fig.7a

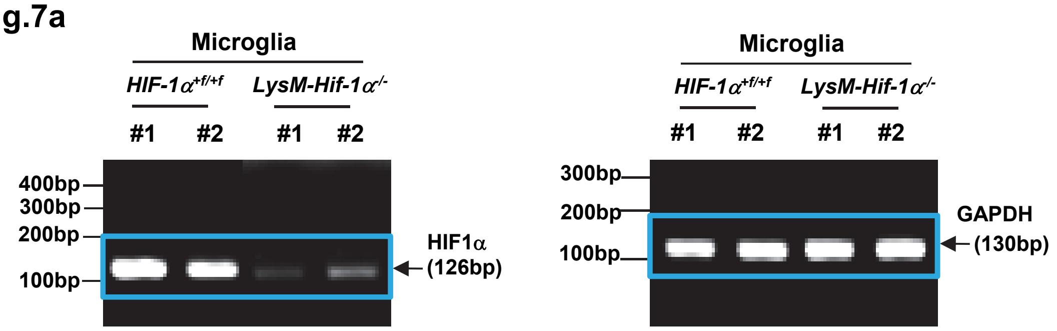

Fig.7b

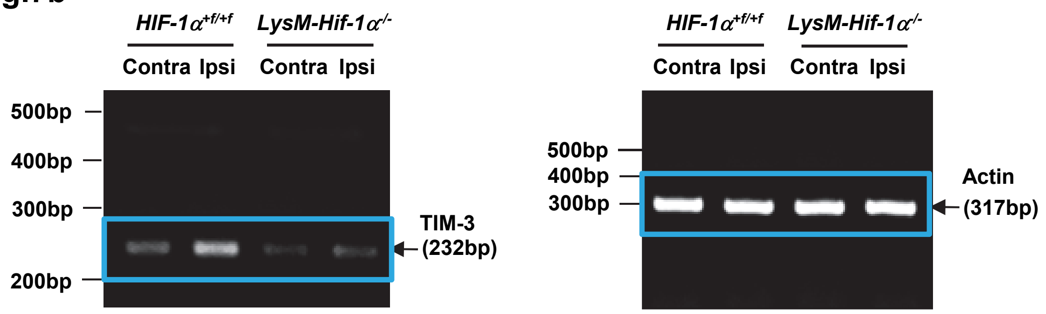

Supplementary Figure 11| **Full scans of blots and gels for Figure 1 (a), Figure 2 (b), Figure 3 (c), Figure 4 (d), Figure 5(e). Figure 6 (f), and Figure 7 (g).**
